# Decision Criterion Dynamics in Animals Performing an Auditory Detection Task

**DOI:** 10.1371/journal.pone.0114076

**Published:** 2014-12-08

**Authors:** Robert W. Mill, Ana Alves-Pinto, Christian J. Sumner

**Affiliations:** MRC Institute of Hearing Research, Nottingham, United Kingdom; Birkbeck College, United Kingdom

## Abstract

Classical signal detection theory attributes bias in perceptual decisions to a threshold criterion, against which sensory excitation is compared. The optimal criterion setting depends on the signal level, which may vary over time, and about which the subject is naïve. Consequently, the subject must optimise its threshold by responding appropriately to feedback. Here a series of experiments was conducted, and a computational model applied, to determine how the decision bias of the ferret in an auditory signal detection task tracks changes in the stimulus level. The time scales of criterion dynamics were investigated by means of a yes-no signal-in-noise detection task, in which trials were grouped into blocks that alternately contained easy- and hard-to-detect signals. The responses of the ferrets implied both long- and short-term criterion dynamics. The animals exhibited a bias in favour of responding “yes” during blocks of harder trials, and *vice versa*. Moreover, the outcome of each single trial had a strong influence on the decision at the next trial. We demonstrate that the single-trial and block-level changes in bias are a manifestation of the same criterion update policy by fitting a model, in which the criterion is shifted by fixed amounts according to the outcome of the previous trial and decays strongly towards a resting value. The apparent block-level stabilisation of bias arises as the probabilities of outcomes and shifts on single trials mutually interact to establish equilibrium. To gain an intuition into how stable criterion distributions arise from specific parameter sets we develop a Markov model which accounts for the dynamic effects of criterion shifts. Our approach provides a framework for investigating the dynamics of decisions at different timescales in other species (e.g., humans) and in other psychological domains (e.g., vision, memory).

## Introduction

Sound, and other sensory inputs, convey information about the environment that an organism can exploit to its advantage. However, to make good decisions about actions the organism must consider factors other than the immediate sensory input. The meaning or value of sounds can vary. As a consequence, an organism must adjust its behaviour to match changes in its surroundings.

In the laboratory, during psychophysical tasks we are often interested primarily in measuring how sensory factors influence decisions. Nevertheless, a variety of non-sensory experimental factors influence subjects’ decisions in detection and discrimination tasks. Such factors include perceptual learning [1], the stimuli used and their statistics [2], [3], [4], and the distribution of rewards [5], [6]. This presents a challenge for psychophysical studies, because decisions cannot be taken as a direct measure of sensation; but it is also an opportunity to study the processes of decision making.

Signal detection theory (SDT) provides a quantitative framework to distinguish sensory and non-sensory aspects of perceptual decisions [7], [8]. Classically, a detection task is formulated as a process in which noisy sensory input is collapsed into a single decision variable, which is then compared to a criterion value to yield a decision. In psychophysics, the stimuli are chosen, the decisions of a subject are measured, and the parameters of ideal (e.g., normal) distributions are estimated, in order to explore the decision-making capacity of the system [9].

The classical SDT approach is to assume that the decision criterion value is static over the period of measurement. Clearly, a single SDT model with fixed parameters cannot account for any non-sensory changes in the way decisions are made over time. This is inconsistent with a large body of experimental evidence for the presence of serial correlations in the responses to independent trials in both human and animal studies [10], [11], [12]. A further observation is that changes in decision processes can occur over multiple timescales. Criterion changes can also be observed across different blocks of data as shifts in response bias. These can be spontaneous, for example accompanying perceptual learning [13], but are often experimentally manipulated via changes in the value of decisions or the likelihood of them being correct ([3], [14], [15], [16], [17], [18], [19], classical animal work). Although the observation of non-stationary decision making in psychophysical experiments is probably as old as psychophysics itself [20], [21], there have been few attempts to integrate what Green [22] termed “molar” psychophysics (the analysis of aggregates of trials) with “molecular” psychophysics (the analysis of each trial separately). There is no standard methodological framework for relating individual decisions in psychophysical experiments to SDT.

Dynamic decision making is not limited to conditions of sensory uncertainty. It also emerges where other task contingencies are uncertain or variable, for example, when the problem is to repeatedly estimate the likeliest value of a noisy quantity (e.g., a stock market forecast, or the probability of a reward given a choice in a laboratory task), where the statistics of the quantity can also change at random points in time. The problem of how to judge whether outcome variability reflects a change in underlying statistics or stationary noise is one that humans [23] and other animals [24] solve nearly optimally. Such adaptive dynamics can be captured by Bayesian ideal observer models that are able to adapt at different rates depending on how expected observations are [23], [24], [25]. Direct neural implementation of Bayesian algorithms would be complex and computationally demanding. However, it has been found that much simpler algorithms, based only on recent (local) observations, also provide near optimal solutions for adapting to task changes over longer timescales [23], [26], [27], [28]. Such algorithms would predict that criterion changes seen at both short and long timescales in psychophysical tasks could arise from a common dynamic criterion setting mechanism. In the current study, we attempt to bridge the gap between classical SDT and dynamic decision making using a computational model. Whilst its generality is unproven, our approach may constitute a framework which can be adapted to many types of psychological experiments (e.g., other species, visual psychophysics, or memory).

The immediate background to the current study is a previous series of psychoacoustic experiments in which ferrets performed a simple yes-no auditory detection task [2], which demonstrated robust criterion effects on different timescales. Firstly, the signal level(s) used in a given behavioural session influenced overall response bias during that session. Secondly, the outcome of one trial affected the decision on the next. For instance, following a false alarm, the ferret was more likely to respond “no”, and following a miss, the ferret was more likely to respond “yes”. Alves-Pinto et al. [2] originally proposed a model consisting of two separate adaptive mechanisms to reflect these respective phenomena, one responsible for setting a long-term, “coarse” criterion based on the signal levels used in the experiment, and another for making short-term, “fine” adjustments around this criterion based on recent trial outcomes. Here, we sought to explain these decision criterion dynamics at multiple timescales in terms of an SDT model in which the criterion is adjusted only according to immediately preceding trial outcomes. To do this, we devised a new experimental format and a related model, which are reported in the two halves of this article.

Common to many psychophysical experiments, our previous study [2] used a yes-no detection task protocol with feedback, such that, during a single session, the signal level was either fixed or drawn randomly. The fixed-level format enabled the investigation of level-dependent changes in bias on the time scale of *sessions*, but there was no instance of a change in level to prompt a change in bias. At the opposite extreme, the randomised format had frequent changes in signal level. In the current study we wished to encourage (and observe) systematic changes in bias both across a single behavioural session and from one trial to the next. To this end, we used same yes-no task as before but manipulated the signal level on a periodic basis, switching to a different set of levels after a fixed number of trials. These data demonstrated robust and repeatable trial-by-trial and block shifts in decision criterion. Block shifts were surprisingly rapid, reaching an asymptote within a few trials.

To account for observations of a dynamic decision criterion, numerous variants of the SDT model have been proposed, in which the criterion value is a variable subject to adjustment [1], [2], [3], [14], [29], [30]. In this work, we assume that the criterion is shifted either upwards or downwards on the basis of the outcome of a trial. If a single trial outcome drives a shift in bias, then it is conceivable that many trial outcomes substantially change the bias over longer time periods. Furthermore, as trial outcomes are a function of signal level, it is plausible that long-term, level-dependent changes in bias actually result from the accumulation of short-term adjustments. In this respect, our model is Markovian and resembles the additive model of Kac [30], but it differs in that it includes a lapse term [31], an exponential (geometric) decay to a steady resting criterion [29], and multiple, blocked signal levels. We adapted our analysis of the model to explicitly account for the blocking strategy and used maximum likelihood to fit the parameters of our model to the ferrets’ decisions.

The inclusion of a dynamic criterion captured trends in the probabilities of a hit and false alarm, as they varied across repeated blocks and as a function of the outcome of the previous trial. A dynamic criterion also significantly increased the performance of the model in predicting decisions from the ferrets. The parameter sets recovered were similar for five ferrets, possibly indicating a common strategy. It was also similar irrespective of whether the stimulus conditions were blocked or completely randomised. We also tested the adequacy for several reduced-parameter variants of the model, in which the criterion was either fixed, was not permitted to decay, or was memoryless. This demonstrated that in the simplest model which accounted for the data, the criterion shifted away from a fixed bias term according to the previous trial alone, with no memory of the outcome of earlier trials. Despite this simplicity, the model was able to account surprisingly well for block-level as well as trial-by-trial criterion shifts. This also supports the notion that the ferrets employed a very simple rule to dynamically adjust their decision criteria, and in this way optimized to some degree their responses according to longer term stimulus statistics.

## Empirical Methods

In this section, we report the methods used to collect, analyse and present data from the behavioural experiments.

### Ethics statement

All procedures were carried out under licence from the UK Home Office and approved by the ethical review process at the University of Nottingham.

### Subjects

Five adult pigmented ferrets (*Mustela putorius*) (three females) were trained and tested in this study. The ferrets were housed individually with environmental enrichment and were permitted daily to interact socially with other ferrets. Behavioural sessions lasted for up to one hour and were typically scheduled to take place twice a day over a course of 11 days. Water bottles were removed from the cages on the evening prior to the first day and returned on the evening of the last day. In the meantime, most of the ferrets’ water intake was supplied by the experimental apparatus to reinforce behaviour. Ferrets were also fed ground ferret food mixed with additional water and a nutritional supplement (Cimicat, Petlife International Ltd., UK) in the evenings. Following the 11-day period, water was provided *ad libitum*, for at least 3 days. Training and testing was discontinued if an animal’s weight dropped 20% below its pre-regulation weight, or if there were any other health concerns.

### Behavioural Apparatus

**Figure 1:**
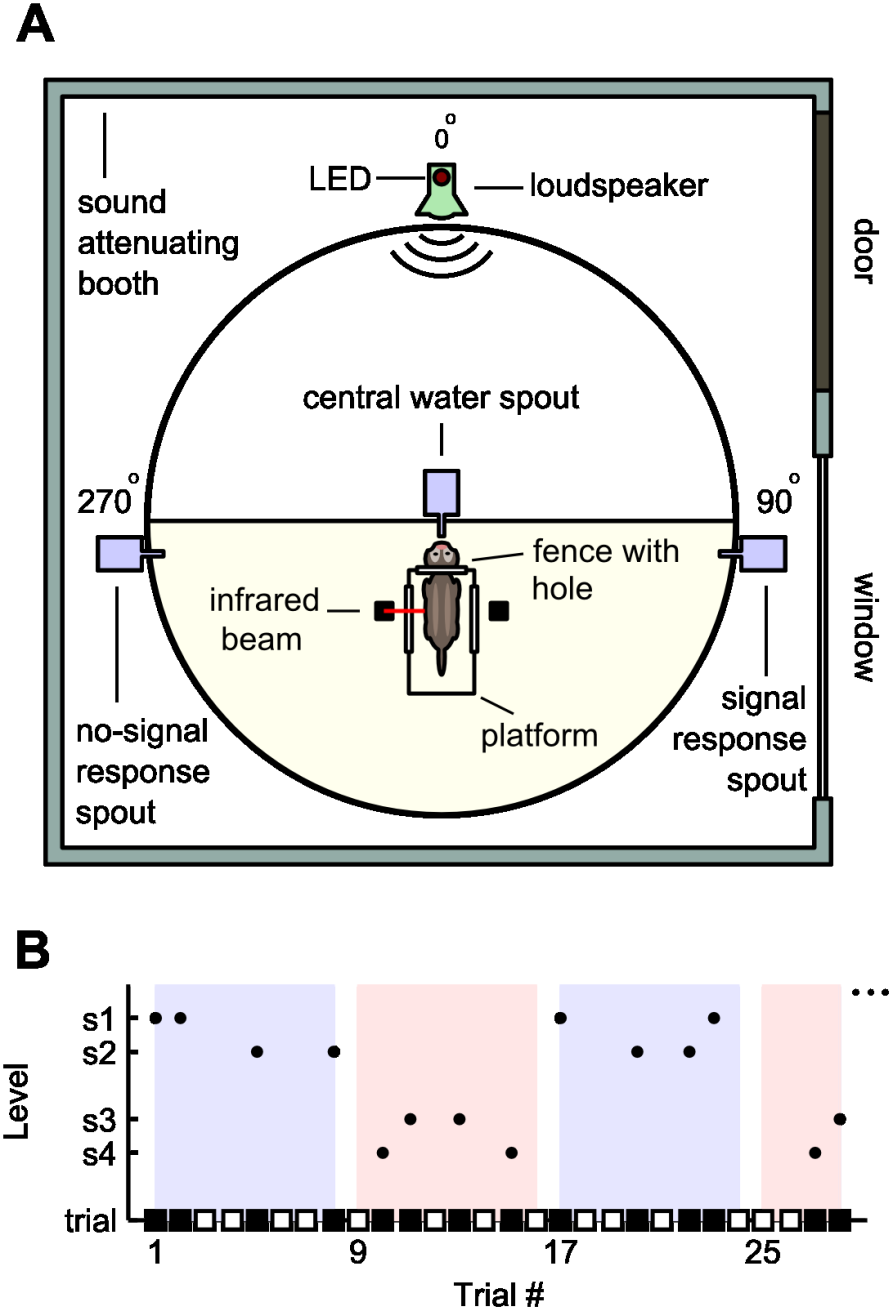
Schematic illustrations of the experimental apparatus and an alternating block stimulus. A) The apparatus used in behavioural experiments. The ferret moved freely in the lower semicircular region of the arena. B) Example showing trials presented in alternating blocks of 8 trials with easy (blue) and hard (pink) detection conditions. Each block contains four no-sound trials and four sound trials (unfilled and filled squares, respectively), and two tones at two stimulus levels (dots, easy: s1, s2; hard: s3, s4). For blocks of 24 trials, there are 12 sound trials in a block, 6 at each of two levels.

Experiments were conducted in a custom-built arena inside a double-walled, sound attenuating chamber (IAC-1204, UK; Fig. 1A). The arena floor consisted of a polyvinyl chloride (PVC) disc (1.5 m diameter). A ceiling, perimeter wall and centre partition were constructed from wire mesh, allowing the ferret to roam freely in one half of the arena. The perimeter was surrounded by acoustically transparent net fabric that concealed custom-made modules. All sound stimuli were delivered via a loudspeaker (Visatron FX10, 70 Hz–22 kHz) encased in a module at 0°. An LED was also mounted on this module to provide a visual signal to the ferret. In the centre of the arena was a platform, three sides of which were closed off by a metal fence. The fence facing the loudspeaker contained a hole, through which a ferret could push its head and lick a central water spout. A lick detector in the spout and an infrared sensor across the platform ensured a consistent head position during stimulus presentation. Responses were recorded (and selectively rewarded) by water spouts with infrared lick detectors attached to modules at 90° and 270°, either side of the centre platform. All three modules were controlled by a MOTU 24 I/O system (Mark of the Unicorn, Cambridge, MA, USA), which was in turn driven by a custom software running outside the booth. A custom USB system controlled the amount of water delivered (∼30 µL for each correct response).

### Behavioural Task

Five ferrets were trained to perform a yes-no detection task [2]. A ferret initiated a trial by licking the centre spout. When a trial was triggered, the LED was illuminated for 0.5 s to provide a visual cue. A target tone was presented on half of the trials (“signal trials”) and was absent on the other half (“no-signal trials”). The ferret received water droplets as a reward if it licked the 90° spout on a signal trial or the 270° spout on a no-signal trial. Incorrect responses were not rewarded. Immediately after registering a response, or following a 30 s period, during which there was no response, the ferret could trigger another trial by returning to the platform and licking the centre spout again. A trial was repeated if there was no response, and, in some cases, when the previous response was incorrect (“correction trials”).

### Stimuli

The target signal was a 10 kHz, 500 ms pure tone, ramped on and off with a 20 ms rise and fall time, and the masker was a 30-s white (full bandwidth) noise sample played in a continuous loop. All sounds were sampled at 96 kHz. The sound pressure level (RMS) was measured with a ½-inch B&K 4165 condenser microphone, pointing upwards and occupying the position where the ferret’s head would be when a trial was triggered. Although the signal frequency and masker were held fixed (masker level: 58 dB SPL), the signal levels were configured in one of two ways during a session: *psychometric functions* (random) or *alternating blocks*.

#### Psychometric functions

Psychometric functions were obtained using the method of constant stimuli [32]. Stimulus levels were drawn randomly from a predetermined level set, chosen to sample the performance range of the ferrets, i.e., from near-chance to near-best. Appropriate ranges were established on the basis of pilot studies and the ferrets’ performances in earlier experiments [2]. For all psychometric functions collected, the probability of a signal trial was 0.5 and correction trials were included.

The psychometric functions presented in this article express the probability of a correct decision for an unbiased observer [9] for a given signal level *s*:
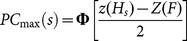



Here *z*(*H_s_*) denotes the *z*-score of the hit rate measured for signal trials at level *s*, *z*(*F*) denotes the *z*-score of the false-alarm rate measured for all no-signal trials, and Φ[·] is the normal cumulative distribution function. The outcomes of correction trials were excluded from the analysis of hit and false alarm rates.

The motivation for collecting psychometric functions was twofold. Firstly, it led to a principled selection of level sets to use in the alternating block paradigm (described below). Secondly, frequent collection of psychometric functions enabled us to confirm a degree of stability in the ferret’s performance. Furthermore, the incorporation of correction trials and a higher proportion of easily-detectable levels during psychometric sessions both reinforced training and encouraged behaviour. Consequently, the first three days (six sessions) of an 11-day experimental period were typically devoted to the collection of psychometric functions.

#### Alternating blocks

In order to determine how the threshold criterion of a ferret changed according to the recent history of decision outcomes or stimulus statistics, behavioural experiments were carried out, in which blocks of trials alternated between “easy” and “hard” level sets (Fig. 1B). The number of trials in a block was a session-level experimental parameter set to either 8 or 24 trials. Within a given block, one half of the trials were signal trials, and the other half were no-signal trials. Of the signal trials, the two levels in a level set appeared with equal frequency, but only one level was presented per trial. The signals and levels were randomised by permuting their position within a block (as opposed to independent sampling with replacement).

Each level set contained two levels, which were chosen with reference to a psychometric function. In our initial experiments, the level sets were spaced by a visual inspection to cover the range of a ferret’s psychometric function. In later experiments, the levels were derived from the psychometric functions using a fitting procedure (see below, “Data Analysis”).

### Data Analysis

#### Fitting regression curves to psychometric data

A psychometric function was measured for a ferret, and the *PC*_max_ values were fitted with a sigmoid function in the form
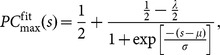
where *µ* and *σ* set the midpoint and slope of the sigmoid, respectively, and *λ* is a lapse probability, i.e., the asymptotic performance at high signal levels. The fit was achieved by minimising the mean square error using fminsearch in MATLAB.

#### Choosing level sets from psychometric curves

Four levels were uniformly spaced on a decibel scale, such that the lowest level, satisfied 
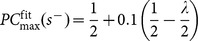
 and the highest level, *s^+^*, satisfied 
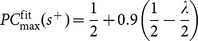
. (See “Method of constant stimuli” above). The joint level set thus spanned the centre portion (∼80%) of the psychometric curve, with the highest and lowest level pairs forming the easy and hard level sets, respectively.

#### Fitting regression curves to blocked data

The probability of a hit and false alarm were measured for each position within in a block, by wrapping around pairs of easy and hard blocks. For example, for sessions with 8-trial blocks, the hit probability for the first trial of an easy block was derived by measuring the proportion of positive responses given on signal trials that fell at positions 1, 17, 33, …, 16*n*+1. Similarly, the false alarm probability for the first trial of a hard block was derived by measuring the proportion of positive responses on no-signal trials that fell at positions 9, 25, 41, …, 16*n*+9 and so forth.

To better estimate the decay time of transitions in these quantities between blocks (in trials, rather than real time), a simple regression was used to fits curves with functional form 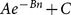 to 4 sets of data points, namely, the hit and false alarm measurements for easy and hard blocks. (This gives a total of 32 data points in the case of 8-trial blocks). The fit was achieved by minimising the mean squared error over all points simultaneously, with the constraint that *B*, the decay constant, be identical for all four curves. Curves fitted to the probability of a positive response or a correct response were derived secondarily from those fitted to hit and false alarm probabilities

#### Receiver Operating Characteristic (ROC) curves

Hit and false alarm probabilities are the two components used to locate detection performance on ROC coordinates. Block-wise changes in bias are revealed by significant relocations of a point in ROC space, dependent upon the trial’s position within the block. To explore more local effects, we also examined data in ROC space conditional upon the outcome of the immediately preceding trial. Thus, in addition to a marker relating the probability of a hit and false alarm on a general trial, we obtained four extra markers relating performance trials preceded by a hit (“yes” in response to a signal), a miss (“no” in response to a signal), a false alarm (“no” in response to no signal), or a correct rejection (“no” in response to no signal).

## Empirical Results

### Alternating Blocks of 24 Trials

The first series of alternating block experiments used blocks of 24 trials and was conducted with ferret 1 as a subject. The purpose of this experiment was, first of all, to confirm that block-level dependencies were actually present in the hit and false alarm probabilities, and, secondly, to identify the time scale of the transitions between blocks (i.e., many trials or few).

**Figure 2:**
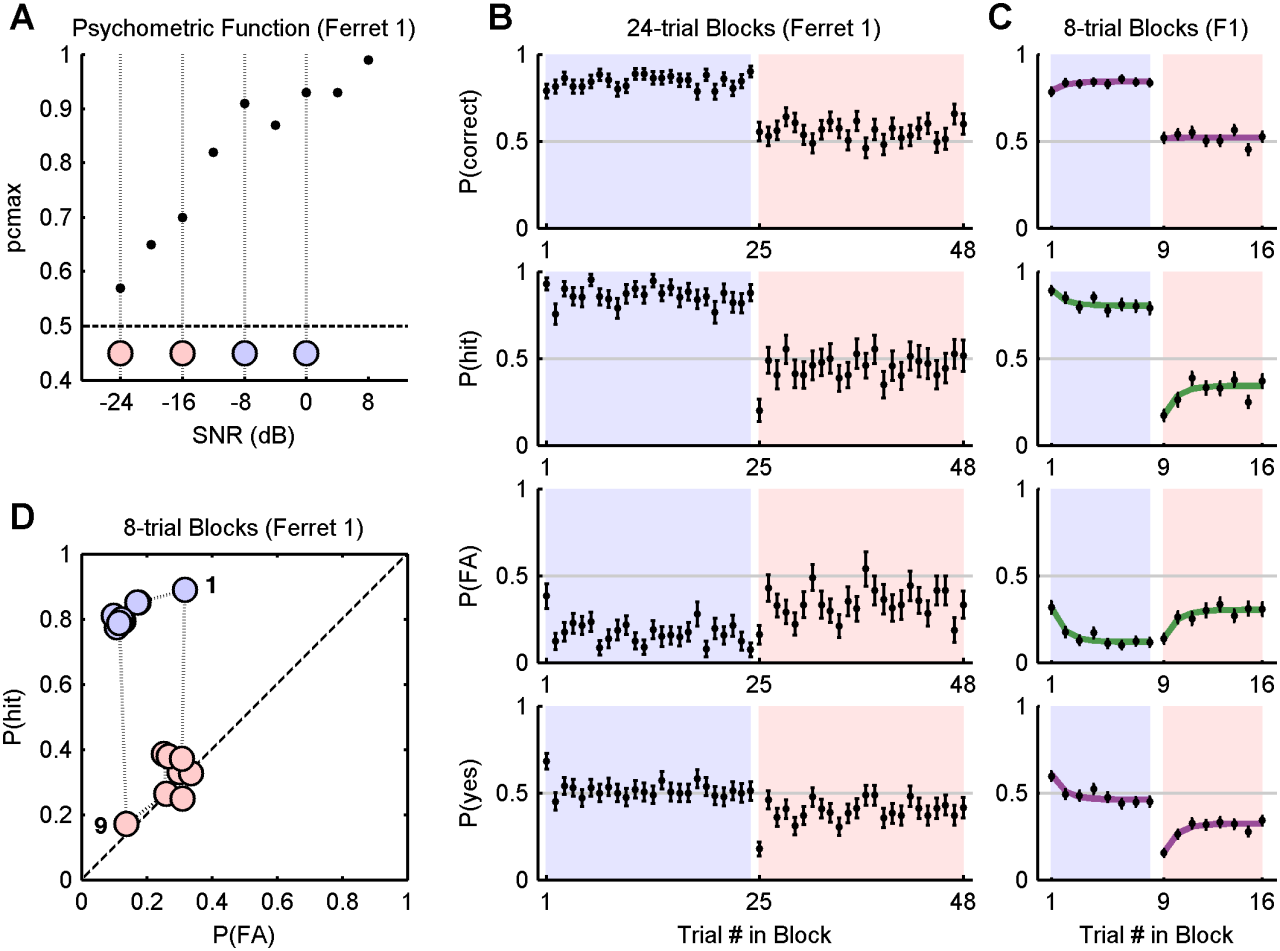
Alternating block task results for ferret 1. A) A psychometric function measured over 6 sessions (dots), expressed as a function of attenuation. Attenuations corresponding to the easy and hard signal levels are marked as blue and pink discs, respectively, and grey verticals. The dashed line corresponds to chance performance. B) Mean outcome of a trial conditional upon its position within an odd or even block of 24 trials. Trials separated by 48 positions are analysed together. Easy trials comprise the odd blocks (blue), hard trials the even blocks (pink). Rows: 1. probability of a correct response; 2. probability of a hit; 3. probability of a false alarm; 4. probability of a positive response. Error bars show s.e.m. C) Mean outcome of a trial as a function of its position in an odd or even block of 8 trials. Trials separated by 16 positions are analysed together. Exponential fits to the data are shown as thick green and magenta curves (see body text). D) Hit and false alarm probabilities from the 8-trial block data (panel C, rows 2 and 3) plotted in ROC space. The fine dotted line is a trajectory linking neighbouring positions, including 16 back to 1. The dashed line corresponds to chance performance. Markers are filled according as they represent as easy (blue) or (hard) trials. The markers corresponding to the mean outcome of the first trial in an easy or hard block are especially highlighted, using “1” or “9”, respectively.

The first six sessions were used to collect a psychometric function. Four stimulus levels were then chosen by inspection to uniformly span the operating range of the psychometric function, corresponding to signal-to-noise ratios of {–24, –16, –8, 0} dB, as shown in Fig. 2A and described in the Methods. These were then divided into an easy level set, comprising SNRs {–8, 0} dB, and a hard level set comprising SNRs {–24, –16} dB. The odd blocks were based on the easy level set. Subsequently, 38 experimental sessions were used to perform alternating block experiments. A maximum of 8 blocks (192 trials) were presented per session. Ferret 1 reached this limit in 4 sessions.

#### Block-level effects

Fig. 2B summarises the results from the alternating blocks experiments. All four rows plot the empirical probability that a trial elicited a certain type of outcome, conditional upon its position relative to the start of the most recent block of easy trials. The statistics of trial outcomes are computed by wrapping the trials from all sessions around two representative easy and hard blocks.

The first row of Fig. 2B plots the probability of a correct decision, *P*(correct). As expected, decisions were more frequently correct in the easy blocks than in the hard blocks, and the specific proportions of decisions that were correct were consistent with the psychometric function, given the choice of level sets (cf. Fig. 2A). The second and third rows plot the empirical probabilities of a hit and false alarm, respectively. The probability of a hit, *P*(hit), was significantly lower during the hard blocks (pooling all signal trials in odd and even blocks: chi-square test, *p*<0.001), and, conversely, the probability of a false alarm, *P*(FA), was significantly higher during the hard blocks (chi-square test, *p*<0.001). The shift in *P*(FA) is the most revealing, because, being calculated from the outcome of no-signal trials, it is independent of the level sets used in the blocks. (The same proposition does not hold for the hit rate, which is calculated from the outcomes of signal trials). Any change in *P*(FA) must therefore have arisen from a difference in decision criterion between then easy and hard blocks. The probability of a yes response is plotted in the fourth row. During the hard blocks, the probability of responding yes was lower. In signal detection terms, Fig. 2B is consistent with a conservative shift in the detection criterion during easy blocks (that is, a positive shift, making “no” decisions more probable).

The results from this preliminary experiment confirm a robust alternation in the mean false alarm rate between easy and hard blocks, establishing that the ferret utilises a variable detection criterion. They also reveal that adaptation is rapid, and occurring within only a few trials. However, for a given session, a limit of eight 24-trial blocks allowed the measurement of only 7 block-boundary transitions. To better focus the data collection around the transitions, a second series of experiments was conducted using 8-trial blocks.

### Alternating Blocks of 8 Trials

The second series of alternating block experiments used blocks of 8 trials and was conducted with ferret 1 as a subject. A maximum of 15 blocks (120 trials) were presented per session, enabling the measurement of 14 transitions (7 easy-to-hard; 7 hard-to-easy). The series ran for 50 sessions.

#### Block-level effects

The results shown in Fig. 2C are formatted in the same manner as those in Fig. 2B. The same significant shifts in the block-level means were observed (chi-square test, *p*<0.001). The data also contain the same rapid transitions between blocks; however, the greater volume of measurements reveal the exponential rise and fall more definitely. Exponential curves were fitted to the hit and false alarm measurements for easy and hard blocks (see “Data Analysis” in Methods), and appear in rows 2 and 3 of Fig. 2C (green curves). This solution has *B* = 1.14, equivalent to a decay time of ∼0.90 trials. Exponential curves describing the probability of a correct decision and a yes response were derived from the hit and false alarm rate parameters, and are plotted as magenta curves in rows 1 and 4.

#### Block-level effects in ROC space

Fig. 2D re-presents the data plotted in rows 2 and 3 of Fig. 2C in ROC space. In this format, the cyclic variation in hit and false alarm probability corresponds to a closed trajectory. There is a sudden jump in hit probability for the first trial in a new block, corresponding to the trials becoming easier (labelled 1) or harder (labelled 9). Changes in difficulty are independent of any putative criterion value, and consequently take immediate effect. On the contrary, the change in false alarm probability lags one or two trials behind–the time required for the criterion to adapt to the levels in the new block.

### Additional Experiments with 8-Trial Blocks

The third series of experiments repeated the 8-trial alternating block paradigm to obtain a larger volume of data with a more objective choice of signal levels. Two ferrets were used (ferret 1 and ferret 2), and signal levels were calibrated more carefully by a uniformly spacing points across the centre of a sigmoidal fit to psychometric data (see Methods). The data collected in these experiments are those used to fit models.

#### Block-level effects

**Figure 3:**
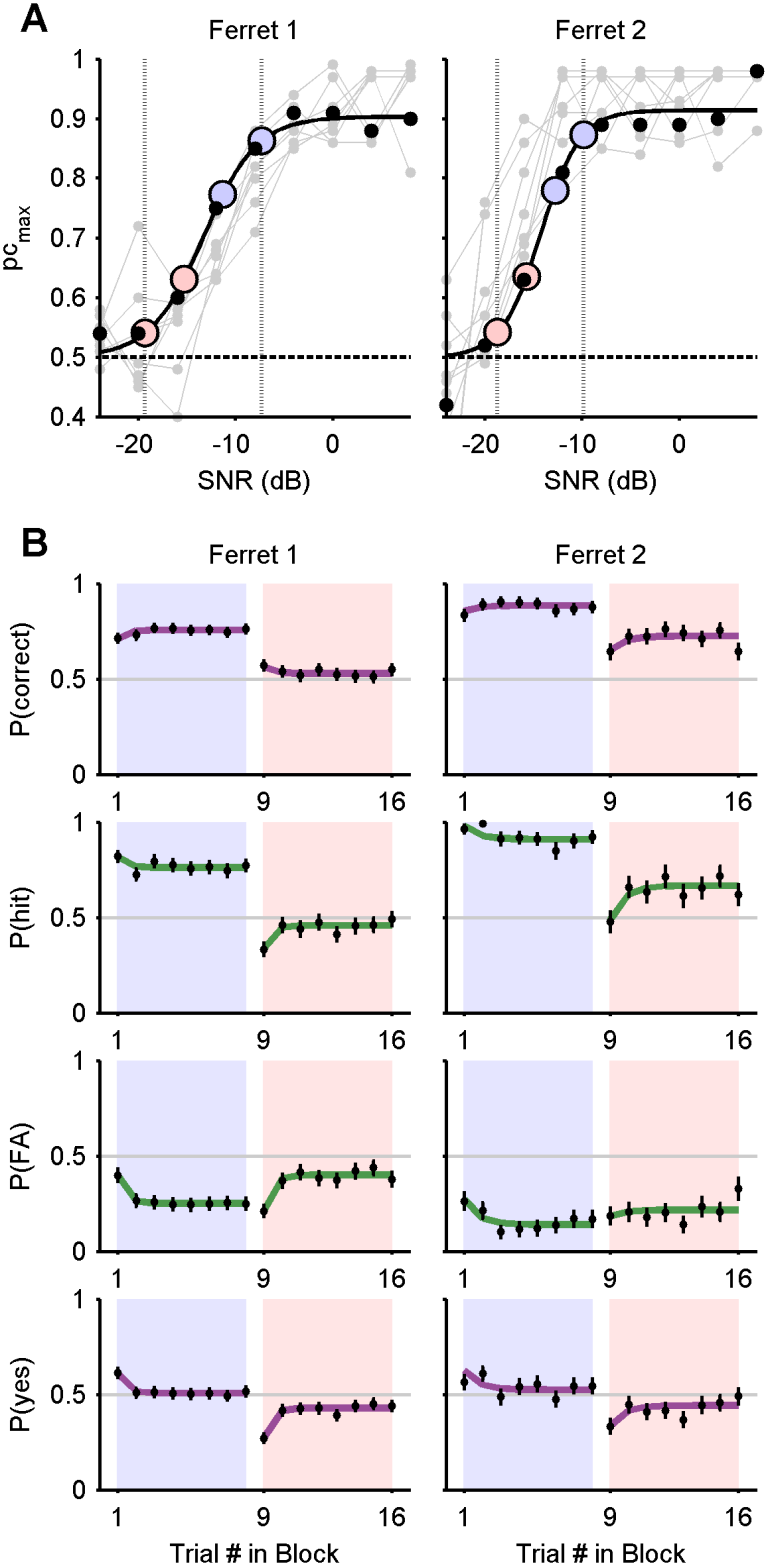
Alternating 8-trial blocks results for ferrets 1 and 2. A) Psychometric functions measured over 6 sessions (dots), expressed as a function of attenuation, and a sigmoid fit (black line). Attenuations corresponding to the easy and hard signal levels are marked as blue and pink discs, respectively. Grey lines and markers show the psychometric functions periodically obtained to monitor performance. B) 8-trial block experiment results (see Fig. 2C).

The level sets and the results of the alternating 8-trial block task are presented in Fig. 3A and 3B, respectively. The ferrets’ responses exhibit the same pattern of exponential rising and falling with a short adaptation time, consistent with that shown in Figs. 2B and 2C. The green and magenta curves were fitted using the same technique (see Methods, “Data Analysis”). The hit and false alarm rates were significantly higher during the easy blocks (chi-square test, *p*<0.001), with the exception of the false alarm rate for ferret 2 (*p* = 0.033).

#### Sequential trial effects

**Figure 4:**
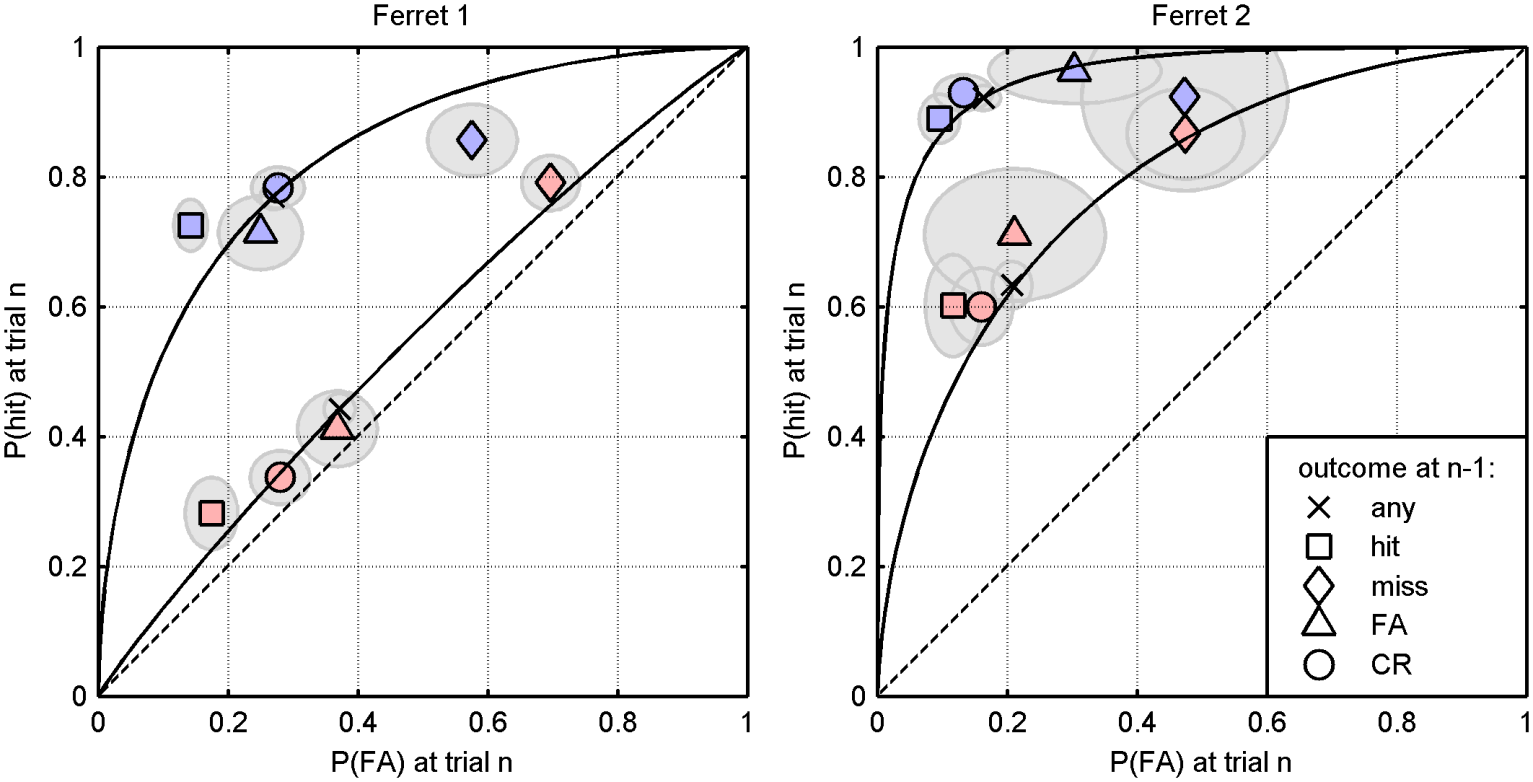
Hit and false alarm probabilities in ROC space. Probabilities of hits and false alarms conditional upon the outcome of a previous trial (see key). The positions of blue and pink markers are computed from trials in easy and hard blocks, respectively, excluding the first trial, which lacks a predecessor. Grey ellipses indicate a 95% confidence interval on the mean. Crosses mark the average probabilities without conditioning. The solid lines passing through them are isosenstivity ROC curves. The chance line is dashed.

Figs. 2 and 3 show that the transitions between blocks are accompanied by an abrupt shift in bias, allowing one to attribute a strong single-trial effect to the first trial in a block, at the least. To visualise more generally the degree to which the outcome of one trial influences the outcome of the next, Fig. 4 plots the probability of hit and false alarm in ROC space, conditioned upon the outcome of the previous trial (marker shape) and the block difficulty (marker colour). For both ferrets, at both difficulty levels, *P*(hit) and *P*(FA) were significantly and sizeably affected by the outcome of the previous trial, as shown by disjoint confidence intervals (95% of the density of a normal distribution). Most notably, misses (false negatives) were significantly followed by the most liberal bias (i.e., in favour of responding “yes”), and hits were followed by the most conservative bias (significantly for ferret 1).

The empirical results demonstrate that the probability of a hit and false alarm varies depending on whether the trial is positioned in an easy or hard block, and the adaptation between blocks is rapid. Ferrets also showed both trial-by-trial shifts in criterion, depending on the outcome of the previous trial. Thus the dependency of criterion dynamics upon both very short and longer term stimulus and response statistics was clearly observable within the same blocked paradigm. The data also reveal that the adaptation to the changes from block-to-block are extremely rapid. The exponential fits in Figs. 2C and 3B give an indication of the time scale of adaptation (time constants ∼1 trial).

## Modelling Methods

We now approach the question of how the criterion dynamics on different timescales might be related. The curves plotted in Figs. 2 and 3 are regressions to summary data, and, as such, are merely descriptive; they do not explain how the curves themselves emerge from individual trials. The strong effect on one trial outcome on the next (Fig. 4) points to a possible mechanism: one in which the criterion is driven by the outcome of the previous trial.

Here we develop a signal detection theory model in which the criterion value drifts over the time, according to the accumulation of trial outcomes. Informally, a reasonable parameter set would discourage errors by shifting the criterion upwards in response to false alarms and downwards in response to misses. We describe the model for the criterion shifts more formally below under the heading “Model Description”. Understanding how to relate these simple rules to behaviour requires us to address two problems.

Firstly, an “analysis” problem arises, in that how likely the criterion is to shift to a particular location depends on outcome probabilities, and these in turn depend on the criterion location. The circularity between parameters and system behaviour are difficult to grasp intuitively, but can be resolved using Markov methods. In the “Model Analysis” section below, we present methods to predict averaged results based on a parameter set.

Secondly, a formal “fitting” problem requires us to find a single parameter set which is able to account for both the change in false alarm and hit probability observed in the data as a function of the position of the trial within a block and the outcome of its predecessor. In the “Model Fitting” section below, we describe a maximum likelihood method to deal with the inverse problem: recovering a parameter set based on the responses given by a ferret over sequences of trials.

### Model Description

The model is a simple signal-detection model, where on each trial some noisy “internal representation” of the sound is compared against a decision criterion, to determine the model’s decision on that trial. After each trial this criterion can shift upwards or downwards a discrete amount depending on the outcome of the trial. In addition, after each trial, the criterion has the tendency to decay a certain proportion of the way back towards a *resting state* criterion.

### Trials

For the *n*th trial in a session (*n* = 1, 2, 3, …), let *h_n_* = 1 if a signal is presented on trial *n*, and *h_n_* = 0 if no signal is presented. Similarly, let *d_n_ = *1 if the ferret responds “yes” on trial *n*, and *d_n_* = 0 if the ferret responds “no”. Then, the outcome of trial *n* is jointly indicated by *r_n_^ij^*, for *i*, *j* ∈{0, 1}, where
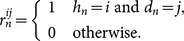



### Signal model

**Figure 5:**
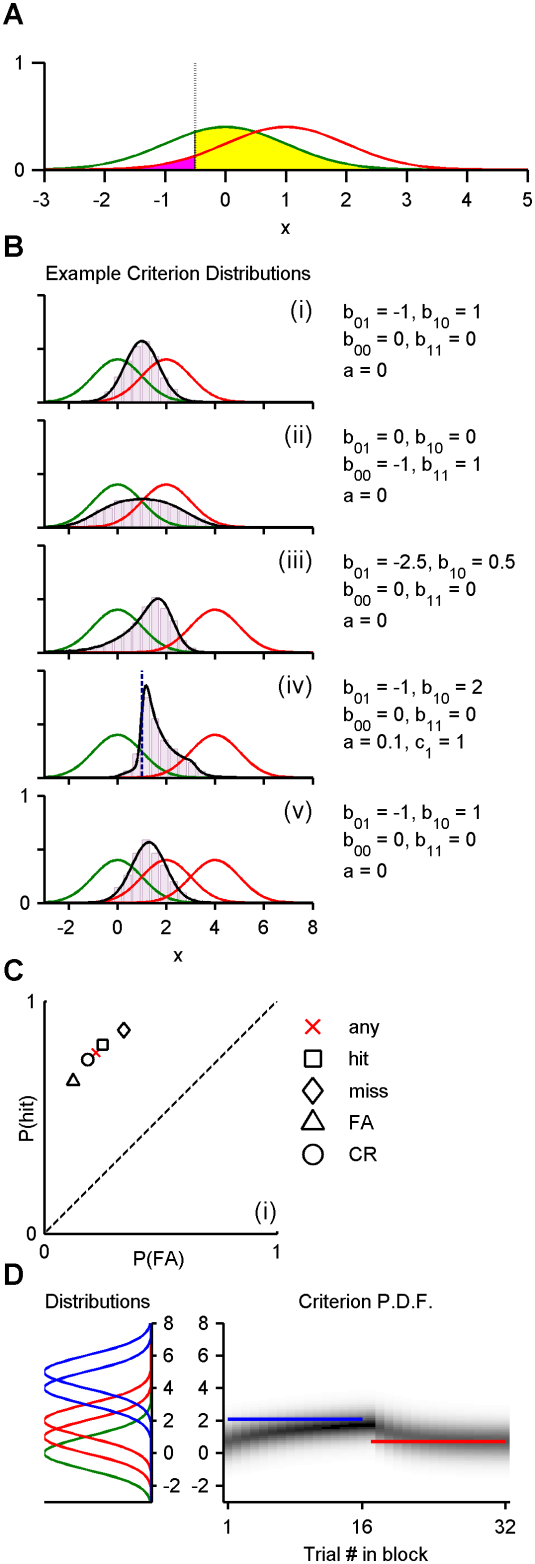
Markov analysis of the criterion shift model. A) Normal probability density functions for no-signal distribution (green curve) and signal distribution (red curve). All distributions have unit variance. Solid areas indicate probabilities of a miss (pink) and false alarm (yellow) given an example criterion position (dotted vertical). B) Stationary criterion distribution (black curve) for four exemplary parameter sets. Criterion histograms obtained from 10,000 Monte Carlo trials are plotted in faint purple. Where it exists, the resting criterion (*c*_1_) is plotted as a dashed blue vertical. C) ROC performance for the model depicted in B(i) conditional upon the outcome of the previous trial (see key). D) Method adapted to analyse alternating blocks of 16 trials. Model parameters are those used in B(i). Left: internal variable distribution, given no signal (green), easy signals (blue), and hard signals (red). Right: p.d.f. of stationary criterion distribution (grayscale) as a function of trial position within blocks. Minimum error criteria are superimposed as blue (easy) and red (hard) horizontal lines. In all examples, the probability of a signal is 50%, and where there are multiple signal distributions, they are chosen from uniformly.

Trial *n* evokes an (unseen) degree of a noisy neural activity, *x_n_*, whose mean directly correlates with the signal level. This quantity is expressed in units on a decibel scale (see below). The variance of the noise is statistically independent of the signal level. On most trials, the ferret’s response is determined by comparing the internal variable to a threshold criterion, *c_n_*. On a minority of trials, the ferret responds randomly (*lapses*). Let *l_n_* indicate a lapse on trial *n*, and λ indicate the probability of a lapse. Then:
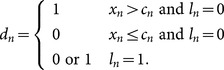



Define *X_n_* to be a normal random variable with standard deviation *σ* and mean *µ_n_*, so that *x_n_* is a realisation of *X_n_*. Here, *µ_n_* denotes the excess in signal level beyond a reference level (dB_ref_), a detection threshold at which the ferret approaches chance performance (see below). Consequently, *µ_n_*≥0, with *µ_n_* = 0 on no-signal trials. Fig. 5A exemplifies distributions for no-signal (*µ* = 0, green) and signal (*µ* = 1, red) conditions.

### Criterion dynamics

The decision criterion applied at trial *n* is designated *c_n_* and is updated on each trial according to the rule
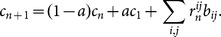



(Note that only one of the response indicators, *r_n_* ^00^, *r_n_* ^01^, *r_n_* ^10^, *r_n_* ^11^, takes the value 1 on a given trial; the rest take the value 0. The summation term therefore corresponds to a single shift, *b*_00_, *b*_01_, *b*_10_, *b*_11_).

The criterion is initially set to a *resting state*, *c*_1,_ at the start of a session. Depending on the outcome of the trial, *r_n_^ij^*, the criterion is incremented by an amount *b_ij_*, where *b_ij_* may assume positive or negative values. The criterion also decays towards the resting state at a rate controlled by *a*, where 0≤*a*≤1, and larger *a* corresponds to more rapid decay. The model is described by a set of eight parameters,
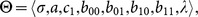



but simpler classes of model can be selected by specialising Θ. For example, fixing λ = 0 disables lapses. If *a = *1, the criterion on each trial is a constant departure from the resting state, *c*_1_, which depends on the outcome of the previous trial. Alternatively, if *a* = 0, the criterion does not drift to a resting state, and *c*_1_ specifies only the initial criterion. Furthermore, setting *b*_00_ = *b*_11_ = 0 specifies a model in which only errors cause criterion shifts.

## Model Analysis

### Monte-Carlo simulations

One can iteratively apply the rules outlined above to randomly sample a decision sequence from a model in response to specific sequence of trials (*h*_1_, *µ*_1_)_,_ (*h*_2_, *µ*_2_), …. First, initialise the criterion to *c*_1_; then, on a trial-by-trial basis, generate the internal variates (*x_n_*, *l_n_*), make a decision (*d_n_*), and derive the criterion at the next step (*c_n_*_+1_) based on the outcome. Following this routine and adopting a Monte Carlo approach, the summary statistics expected for a particular parameter set can be extracted from one, long randomly-generated sequence, and presented graphically (e.g., in the formats used Figs. 2, 3 and 4).

### Semi-analytical Markov solution

An alternative solution to Monte-Carlo simulations is to use a semi-analytical solution, by which we can for any one set of parameters directly calculate the mean model output for a given trial in a given block. This capitalises on the Markov assumption inherent in the proposed criterion update rule, namely, that when the criterion on a trial is conditioned upon the criterion on the previous trial, it is statistically independent of the criteria on all the earlier trials, *p*(*c_n_* | *c_n_*_–1_, *c_n_*_–2_, …, *c*_1_) ≡ *p*(*c_n_* | *c_n–1_*). For example, consider a rule that moves the threshold one unit to the left in the event of a miss (*b*_01_ = –1) and one unit to the right in the event of a false alarm (*b*_10_ = +1). (For a discussion of this model, see S1 Supplementary Material). Then, following Fig. 5A, if the criterion is placed such that the probability of a false alarm (yellow area) exceeds the probability of a miss (magenta area), then a shift to the right is more probable than a shift to the left. As the threshold proceeds upwards, shifts to the left become more probable. In the limit of many trials, a stationary distribution emerges, from which one can directly obtain certain familiar statistics, such as the overall false alarm probability.

We now substantiate this intuition formally. Let *f_n_*(*c*) denote the probability density function of the criterion at trial *n*. One can then write
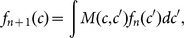



or more briefly, *f_n_*_+1_ = **M** *f_n_*, where **M** ≡ *M*(*c*, *c*′) is a constant kernel that depends both on the stimulus statistics and θ, and assigns a probability density to criterion transitions from *c*′ to *c*. Because **M** is independent of trial position, the evolution of the criterion distribution satisfies the Markov property, and the density function can be iteratively derived for successive trials, i.e.,
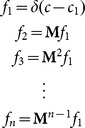
where *δ*(·) is the Dirac delta function. In most circumstances of practical interest, as 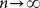, the criterion distribution approaches a stationary distribution *f* *, which satisfies *f* * = **M** *f* * [33].

### Application to blocked stimuli

The Markov approach employed above is predicated on the probability of transitions from one criterion location to another being independent of the trial number. However, in the blocked stimuli experiment, this assumption fails, as *M* depends on the signal statistics, which regularly alternate. The Markov approach can be modified explicitly to account for blocking. If blocks consist of *L* trials, and criterion shifts during easy and hard blocks are governed by *M*_easy_ and *M*_hard_, respectively, then one instead solves
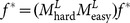



for *f* *, to find the stationary criterion distribution that holds for the first trial of an odd block. From there, appropriate compositions of *M*_easy_ and *M*_hard_ are used to find the criterion distribution as it applies on the 2*L*–1 remaining positions within a cycle. This approach generalises to any repeating cycle of blocks.

### Parameter Fitting

#### Mean squared error model

The previous section outlined how, for a given parameter set, Θ, long-term statistics could be obtained for a repeating block pattern, either by simulating many Monte-Carlo trials, or by solving the stationary criterion distribution, *f* *. In principle, then, one could fit the data by varying Θ and selecting parameters that best match the some statistical aspect of the empirical data. For instance, one might vary Θ to minimise the mean squared error between the empirical curves in **Fig.** 3 and the expected analytical curves, or the mean squared Euclidean distance in ROC space between the markers in **Fig.** 4 and their respective model predictions. We fall back on this latter, brute-force approach when more advanced techniques fail (see discussion of likelihood maximisation below). For this brute force method, the fit quality is measured using the mean Euclidean metric for all combinations of six shift values for the four outcome types (*b_ij_* spaced uniformly between –10 and 10), and six distribution widths (σ spaced uniformly between 3 and 12): a total of 7776 ( = 6^5^) combinations. The best solution on this “coarse” grid is then refined by testing parameter choices on a “fine” grid, formed by subdividing the best grid cell another 6 times for each parameter (i.e., another 7776 sub-cells). Thus, the candidate solution space has a granularity in which *b_ij_* points are **≈**0.5 apart, and σ points are **≈**0.25 apart.

#### Likelihood model

A more principled variation on this approach seeks to account for the raw observations first, that is, the sequences of actual decisions. Given the model description above, the probability of a “yes” decision on trial *n* of a session is given by
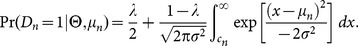



Considered in isolation, the criterion on a given trial, *c_n_*, is a random variable. However, the expression *d_n_* depends on the variate, *c_n_*, which is determined by trial *n*–1, as it depends on previous outcomes (see recursive for *c_n_* above).

Abbreviating the stimulus sequences up to trial *n* using 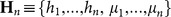 and the decision sequence up to trial *n* using 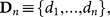 the log-likelihood of the parameters Θ given the entire sequence of *N* trials is
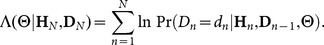



Maximising this expression constitutes a method for fitting a model to individual decisions. Thus, it does not explicitly fit the model to the data points (hits, false alarms) in Figs. 2–4. Rather, any resemblance of the model to these plots is a consequent of a degree of agreement with individual decisions the ferret made.

#### Maximising the Likelihood

If Θ*^i^* is an estimated parameter set, then ascending the gradient of the likelihood function a small amount, *η*, gives an improved parameter set,
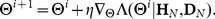



The partial derivatives of Λ with respect to the components of θ are fully derived in S2 Supplementary Material.

The decision sequences from the 8-trial alternating block sessions for ferret 1 and ferret 2 were separately fitted using stochastic gradient ascent [34]. The fitting procedure was carried out was as follows. Parameters were initialised to sensible initial values (the parameter shifts were set to zero). Sequences recorded in experimental sessions were drawn randomly without replacement, and a single gradient ascent step was performed to improve the parameter set on each iteration. Once the total set of sessions was exhausted, the procedure resumed again with the full set, taking each session in turn. One hundred iterations were carried out with a coarse update step, η = 0.1, holding the lapse probability fixed at λ = 0.05. Two thousand iterations were then performed with a finer step sizes (η = 0.01 for 1000 steps, η = 0.001 for 1000 steps), now allowing the lapse probability to fit freely. Convergence was verified visually. The rationale for fixing the lapse probability on the first 100 iterations was to enforce a reasonable initial fit to the data, and only later to allow exceptions to constitute lapses. (Otherwise, we found that the lapse probability converged prematurely to λ = 1).

## Modelling Results

The modelling challenges are as follows: does the model adequately account for the criterion shifts observed in the data? What aspects of the model determine its success or failure in predicting the data? Does the model offer insights into to how the trial-by-trial and block level criterion shifts are related? We will begin by exploring overall model behaviour with some example hypothetical parameter settings. We will then consider the adequacy of the full model, before exploring the limitations of several reduced models. Finally we will explore whether the model can also fit more a general psychophysical datasets. At this point, we will be in a position to advance some tentative conclusions about the ferrets’ on-going strategy in performing this task.

### General properties of the model

Different sets of parameters are expected to yield different model behaviour. In the model criterion is a consequence of summing shifts over trials, consequent on the outcome of each trial. Thus even average behaviour is a product of both model parameters and stimulus parameters. The Markov analysis of the model represents an efficient method for determining the stationary criterion distribution and, from that, stationary hit and false alarm probabilities, given a set of model parameters. The parameters are: an internal noise standard deviation (σ), constant shifts in response to the previous trial outcome (*b_ij_*), a criterion decay with a fixed decay (*a*) to a steady criterion (*c*_1_), and a lapse probability (λ). Here we provide some illustrative examples of model solutions based on artificial parameter sets.

### Solutions with identically-distributed trials

Fig. 5B plots the stationary distributions (black) that result from four respective parameter sets. These were calculated analytically from the Markov analysis. In these examples, signal levels are drawn independently on each trial from an identical distribution (or set of distributions); there is no blocking. Note that a small amount of noise is added to each criterion shift (normal distribution, zero mean, standard deviation 0.01).

In 5B(i), the criterion is shifted by a unit amount in the direction opposing the error. The criterion remains fixed after a correct response. This illustrates how even very simple criterion shift rules can in principle set decision criterion quite effectively: many false alarms drive the criterion upwards, increasing the probability of misses; as misses become more probable, the criterion is driven downwards, and so on. The resulting equilibrium leads to a criterion distribution that falls symmetrically around the minimum error criterion, even though there is no resting criterion this model. Narrower distributions result from smaller step sizes (results not shown).

In 5B(ii), the criterion is shifted by a unit magnitude in the direction opposing a *correct* decision; that is, the criterion shifts downwards following a correct rejection and upwards following a hit. The criterion remains fixed after an error. The resulting criterion distribution also falls symmetrically around the minimum error criterion and is somewhat wider than that of the error-driven model from Fig. 5B(i), even though the shift magnitudes are equal. This difference can be explained in terms of overall outcome probability. If the criterion is around the optimum, then errors are rare and correct decisions are frequent. Hence, in the model driven by errors, (i), the criterion shifts rarely, so that most of the probability mass is concentrated around the optimum. In the model driven by correct outcomes, (ii), the criterion shifts often, so the probability mass extends over a larger region. A model that *reinforces* correct decisions (*b*_00_ = 1, *b*_11_ = –1) is unstable.

In 5B(iii), criterion only shifts following errors, but criterion shifts are greater for misses than false alarms. Consequently, the criterion distribution is skewed downwards, producing a bias in favour of positive decisions.

In 5B(iv), the criterion continually decays towards a resting value (*c*_1_ = 1), but larger shifts are incurred for false alarms than for misses.

In 5B(v), the signal distribution consists of a mixture containing two levels that occur with equal probability. This sort of two-level configuration governs a single block in the ferret experiments described earlier. Compared with 5B(i), this criterion distribution is very slightly broader, but the mean is also higher. This illustrates further how various criterion shift rules can in principle move the criterion toward an optimal value.

In each of Fig. 5B(i)−(v), the criterion distribution is superimposed on a bar graph showing the histogram for 10,000 Monte Carlo trials. In each example, there is a close match between the analytical and empirical results. (The empirical results converge perfectly to the analytical curves as the number of simulated trials increases; results not shown).

Fig. 5C plots the ROC markers corresponding to trial outcomes for the setup used in Fig. 5B(i), conditional upon previous trial outcome. The markers corresponding to outcomes following errors are displaced from the marker corresponding to any trial (the unconditional probabilities), which is perhaps to be expected, as, in example (i), errors are the outcomes associated with shifts. Despite the fact there are no shifts associated with correct outcomes, the markers for hits and correct rejections are also displaced.

### Solutions with blocks of identically-distributed trials

Fig. 5D shows an example in which conditions alternate every 16 trials between easy levels (left panel: µ = 3, 4; blue) and hard levels (left panel: µ = 1, 2; red), in the same fashion as the ferret experiments described earlier. The shift parameters used are the same as those used in Fig. 5B(i). The right panel plots the criterion distribution as a function of the trial position within blocks. The distribution shifts between two asymptotes as the block statistics change, the mean criterion being more conservative during easy blocks. From this we can see that qualitatively at least, the shifts in block-level criterion can be produced by the model.

### Parameter Fitting Results

#### Fitting the full model to blocked trials

**Figure 6:**
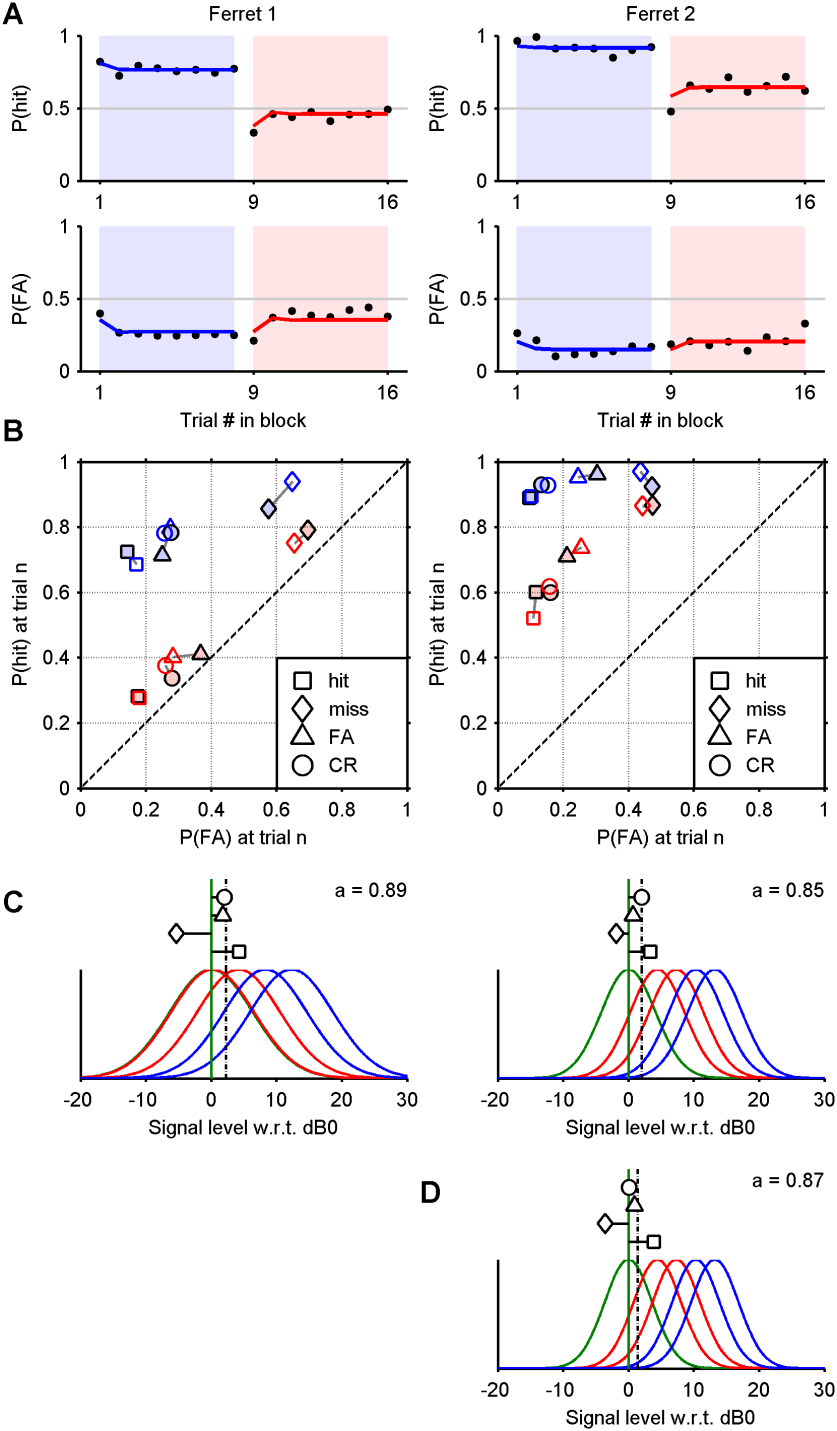
Maximum likelihood fits to data from ferrets 1 and 2. A) Probability of a hit and false alarm given the position of a trial in a block. Empirical data (dots, see Fig. 3); fit from Markov model based on maximum likelihood parameters (solid lines). B) Probability of a hit and false alarm plotted in ROC space given the outcome of the previous trial. Empirical data (solid markers, see Fig. 4); fit from Markov model based on maximum likelihood parameters (hollow markers). C) Maximum likelihood parameters for alternating blocks of 8 trials (shifts *b_ij_*, markers on stems–see key in B; *c*_1_, dashed vertical; no/hard/easy signal densities, green/red/blue curves). D) Maximum likelihood parameters for trials presented in a random order (ferret 2 only).

The fitting procedure adjusts model parameters to maximise the likelihood of full decision sequences for two ferrets in light of the stimulus sequences presented to them; there was no explicit effort to optimise the fit to data points as shown in Figs. 2–4. Fig. 6A, B shows that the parameterised model successfully captures summary performance statistics, such as a probability of a hit and false alarm given the position of the trial within a block (Fig. 6A) and the outcome of the preceding trial (Fig. 6B). The parameter sets for each ferret are depicted graphically in Fig. 6C and display the internal noise (σ, curve width), the four shifts (bij, horizontal stems), the resting criterion (c1, dashed vertical), and the decay parameter (a, inset label). According to both the summary data itself (A, B) and the model parameters that best explain those data (C), the two ferrets exhibit remarkably similar behaviour: the most liberal criterion shifts follow misses and the most conservative shifts follow hits, with the other two outcomes falling in between. The fitted parameters are tabulated in the first two rows of Table 1. In both cases, the resting criterion falls slightly above the centre of the noise distribution, indicating a weak proclivity to respond positively on no-signal trials.

**Table 1:** Parameters fitted to full model.

Ferret	Trial Format	σ	*a*	*c*_1_	*b*_11_	*b*_01_	*b*_10_	*b*_00_	λ
1	Blocks (8)	6.38	0.89	2.30	4.28	−5.28	1.83	2.02	0.07
2	Blocks (8)	4.16	0.85	2.04	3.33	−1.81	0.70	2.06	0.05
2	Random	3.65	0.87	1.44	3.87	−3.51	0.92	0.10	0.06
3	Random + CT	12.36	0.83	7.81	8.25	−1.13	1.81	2.62	0.04
4	Random + CT	10.89	0.96	5.31	6.72	−7.99	6.31	0.97	0.03
5	Random + CT	9.41	0.70	5.63	6.79	−2.60	1.85	2.18	0.04

#### Fitting the full model to unblocked trials

If the ferret truly adopts the criterion shift model we have described, then a procedure that maximises the likelihood of the decisions should yield the same parameter set, regardless of how the trials are arranged. In other words, the fit should not reflect the fact that the trials were organised into blocks. To address question (b), the same maximum likelihood fitting procedure was also carried out for data collected for ferret 2, in which the same signals levels were used but presented in a randomised order (that is, equiprobably, without blocking, and with 50% probability of a signal). The fitted parameters are displayed in **Fig.** 6D and give a qualitatively similar result to that shown in **Fig.** 6C (right): namely, liberal shifts after misses, conservative shifts after hits, and a resting criterion ∼2 dB w.r.t. the reference level. They are also tabulated in the third row of Table 1. These results speak in favour of both the full model and the procedure used to fit it, in that a similar set of parameters is obtained regardless of how the trials are arranged.

The adequacy of the model results thus far support the notion that criterion dynamics driven by the outcome of the previous trial account for not only the trial-by-trial effects, but can also explain with a degree of quantitative accuracy the shifts in criterion across different blocks of stimuli.

#### Fitting a model without criterion shifts

**Figure 7:**
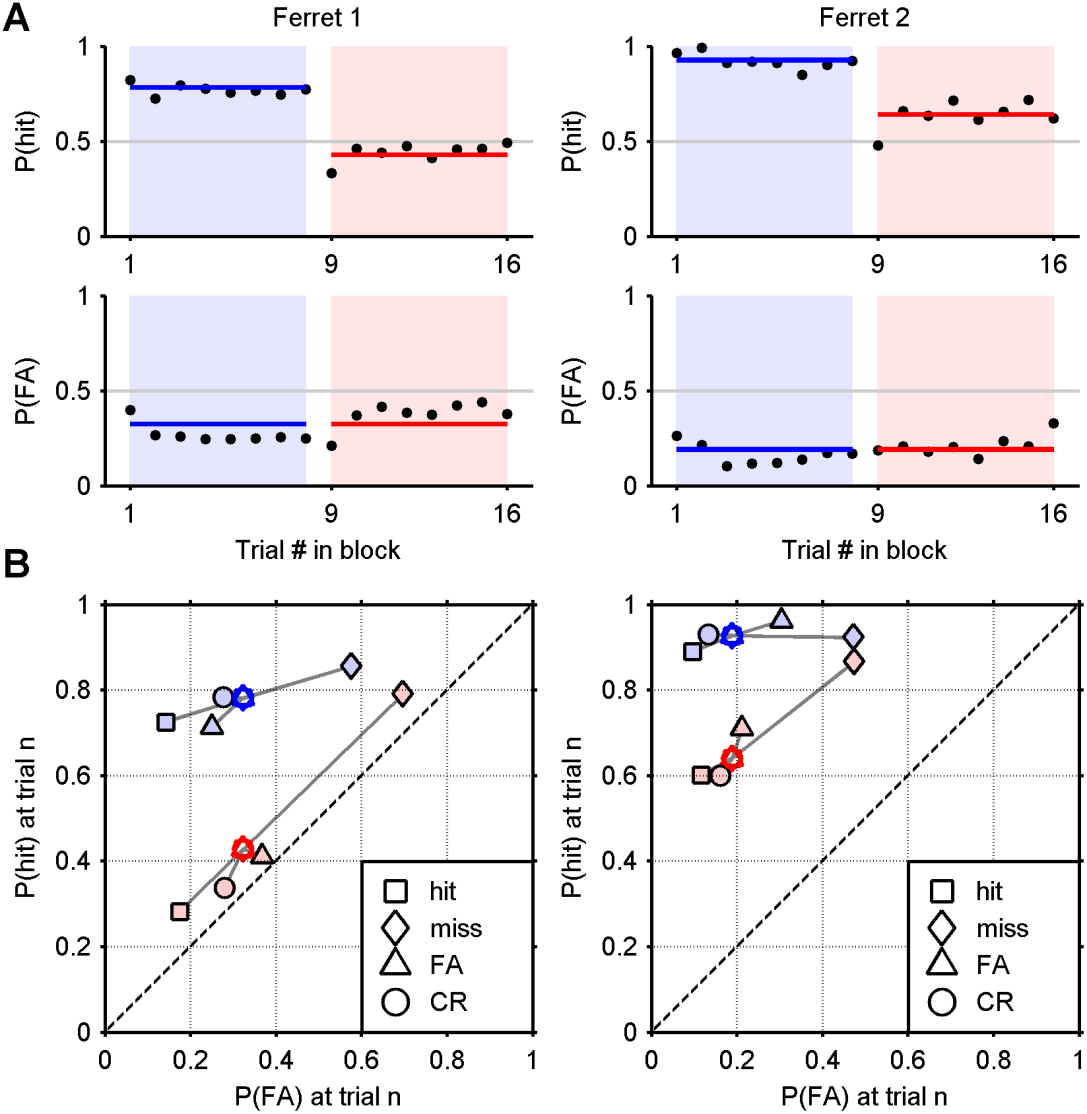
Maximum likelihood fits for an additive model without criterion shifts. A) Probability of a hit and false alarm conditional upon the position of a trial within a block (yellow area: difference). B) Probability of a hit and false alarm conditional upon the outcome of the previous trial (solid markers: empirical; hollow markers: model). The chance line is dashed. Compare Fig. 6.

In order to understand how different components of the model contribute to the fitting of the empirical results, we fitted reduced forms of the model, in which the variety of criterion behaviour was diminished by disabling certain features. In the first instance, all criterion shifts were disabled by setting *b_ij_* = 0 (a “no-shift model”). Under these circumstances, the model is only free to adjust only the distribution widths (σ), the resting criterion (*c*_1_) and the lapse probability (λ). The results are shown in **Fig.** 7, formatted in a manner identical to that of **Fig.** 6A, B. The model performs poorly, when contrasted with the full version. Evidently, the maximum likelihood procedure achieves a first-order approximation: the solution describes a static criterion which is an “average” of the steady criteria during the two blocks. The outcome of one trial naturally does not affect that of its successor, so that four points in ROC space (conditioned on hit, miss, FA and CR) fall at the same point. The parameters fitted to the no-shift model are tabulated in the first two rows of Table 2. Note that the distribution widths are slightly wider, in order to accommodate the absence of criterion shifts.

**Table 2:** Parameters fitted to no-shift model.

Ferret	Trial Format	σ	*c*_1_	λ
1	Blocks (8)	7.79	3.76	0.04
2	Blocks (8)	4.33	0.27	0.06
3	Random + CT	12.04	12.47	0.04
4	Random + CT	11.15	8.05	0.04
5	Random + CT	8.82	9.83	0.04

#### Fitting a model without criterion decay

**Figure 8:**
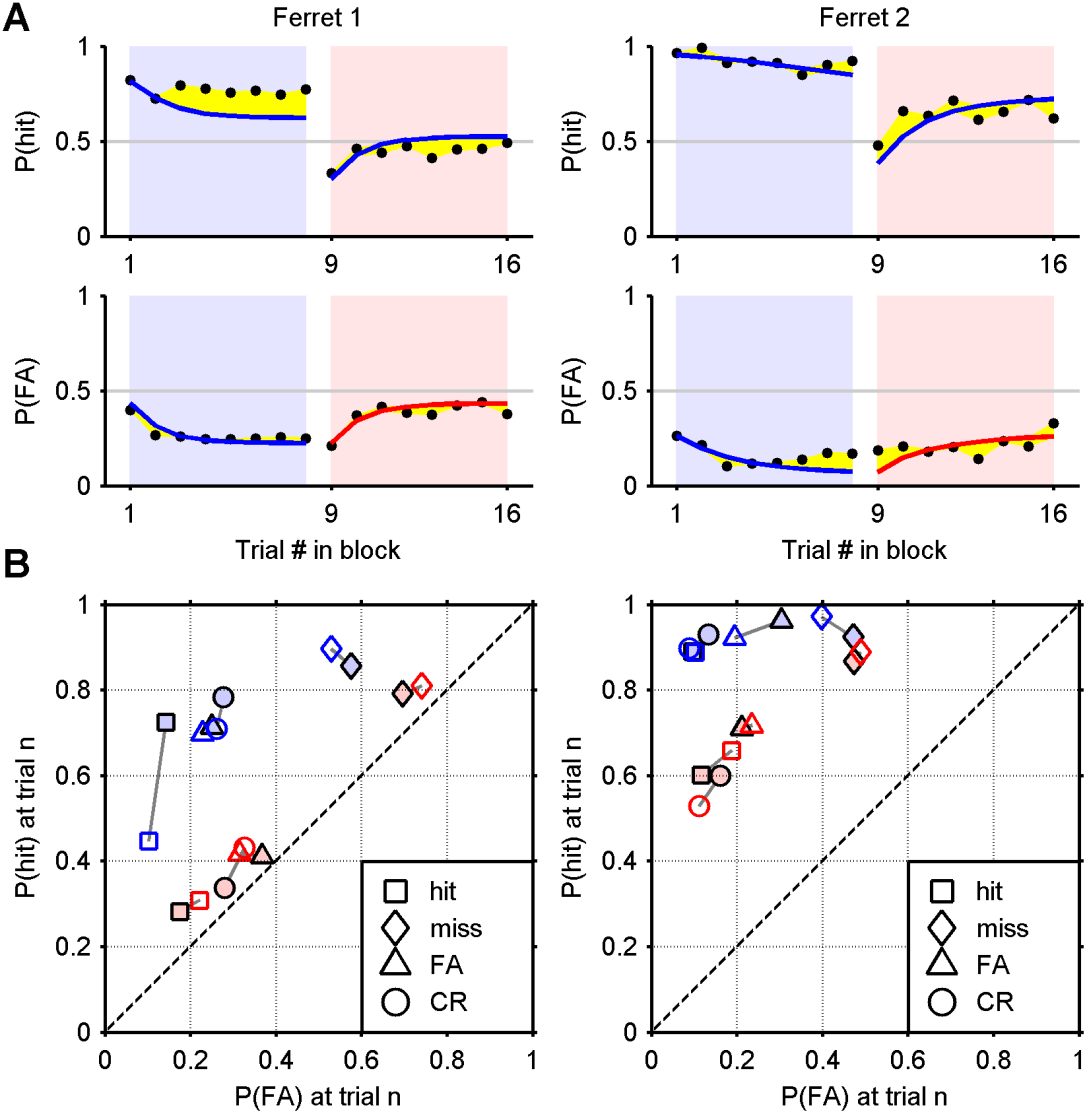
Maximum likelihood fits for an additive model without criterion decay. A) Probability of a hit and false alarm conditional upon the position of a trial within a block (yellow area: difference). B) Probability of a hit and false alarm conditional upon the outcome of the previous trial (solid markers: empirical; hollow markers: model). The chance line is dashed. Compare Fig. 6.

We also evaluated a second variant of the model, in which the criterion was permitted to shift, but there was no decay to a resting criterion (the “no-decay model”). That is, *b_ij_* are free to vary, *a* = 0, and *c*_1_ describes only the *initial* criterion value. Fitting this model using the maximum likelihood technique was impractical, due to the lack of a decay term to stabilise the model. Instead, we used an exhaustive search of a discretised parameter space, to find parameters that adequately fitted the data (see Modelling Methods). The metric used to fit the data was the mean Euclidean distance between the coordinates of the points in linear ROC space that describe conditional outcome probabilities for both empirical and analytical results. (Informally, the parameters were adjusted so as to minimise the average length of the grey lines connecting the hollow markers to the solid ones in **Fig.** 8B). It is important to note that the exhaustive search has an advantage over the maximum likelihood approach in that it is an attempt to fit the summary analysis data directly rather than predicting individual decisions. The results are shown in **Fig.** 8, formatted in a manner identical to that of **Fig.** 6A, B and **Fig.** 7. Although it is clear the no-decay model outperforms the one lacking criterion shifts altogether, it does not perform as well as the model that includes a criterion decay term. The no-decay model manages to capture roughly the shape of the summary data, as presented in block (A) and ROC (B) formats. The fit to the false alarm data for ferret 1 is visually impressive. However, the other curves in **Fig.** A are an imperfect fit, showing a systematic departure from the empirical data in various places (e.g., ferret 1 hit rate in **Fig.** 8A). And, in **Fig.** 8B, some of the markers in ROC space are poorly aligned, most notably, the “hit” marker for ferret 1. Overall, the fit captures qualitatively the main features of the data, but the lack of the decay parameter, *a*, leads to a failure to retain some of the nuances in the data. The parameters fitted to the no-decay model are tabulated in Table 3.

**Table 3:** Parameters fitted to no-decay model.

Ferret	Trial Format	σ	*b*_11_	*b*_01_	*b*_10_	*b*_00_	λ
1	Blocks (8)	5.16	8.40	−12.40	6.80	−2.80	0.05
2	Blocks (8)	2.64	1.20	−6.80	2.80	0.40	0.05

#### Fitting a model with no criterion memory

**Figure 9:**
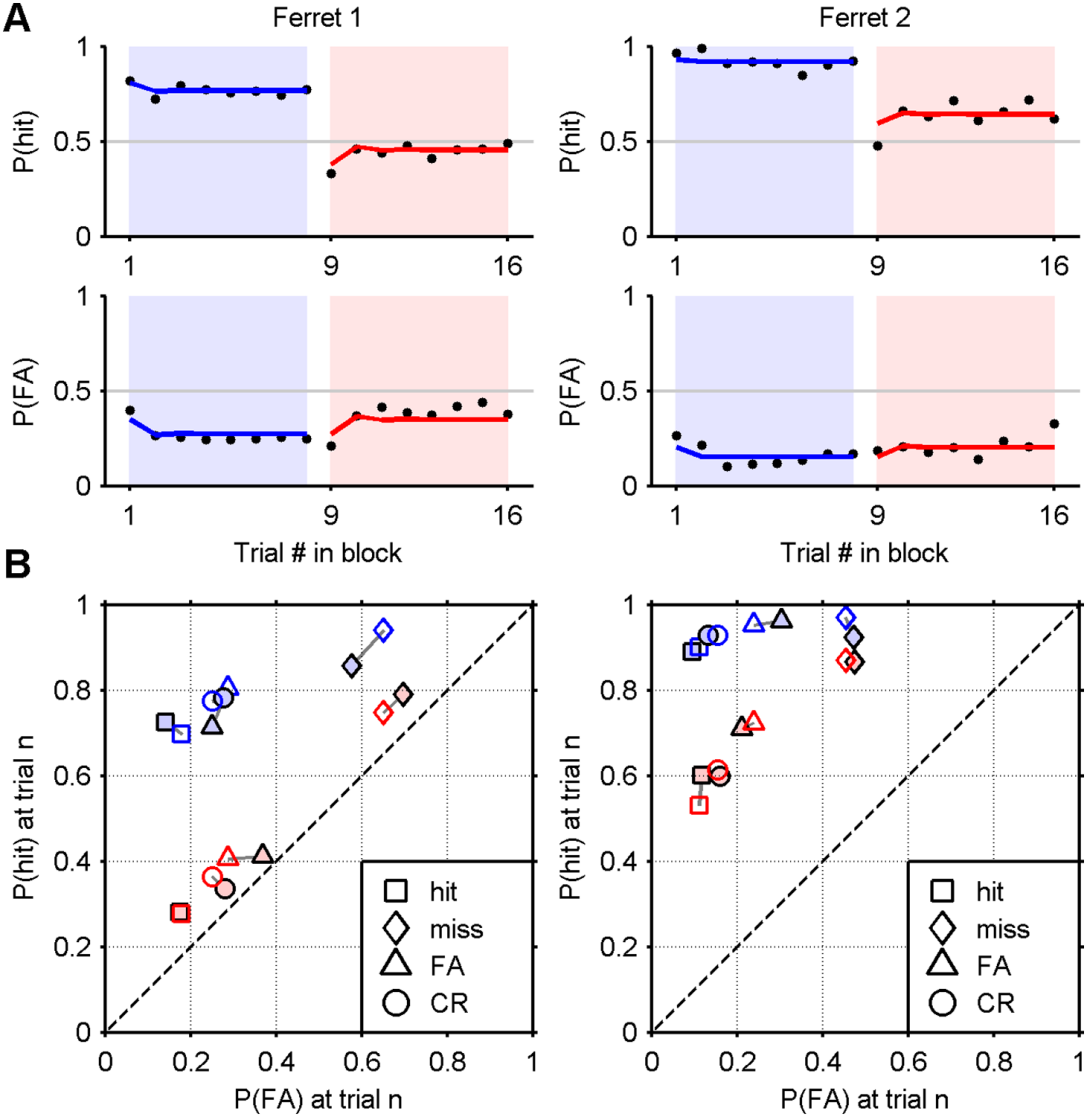
Maximum likelihood fits for a model with no memory of the criterion on the previous trial. A) Probability of a hit and false alarm conditional upon the position of a trial within a block (yellow area: difference). B) Probability of a hit and false alarm conditional upon the outcome of the previous trial (solid markers: empirical; hollow markers: model). The chance line is dashed. Compare Fig. 6.

A third variant of the model is obtained when criterion shifts are only retained for the immediately following trial, that is, *a* = 1 (“no-memory” model). Thus on each trial, the criterion is, *c_n_* = *c*_1_+*b_ij_*, where *i* = *h*_n–1_ and *j* = *d*_n–1_. This might appear to be an overly simple model. However, recall that *a* is close to 1 (0.85 and 0.89) in the fully fitted models (**Fig.** 6). **Fig.** 9 shows the results of the maximum likelihood fit of the no-memory model to the data, which is almost indistinguishable from the full model (see Table 4 for parameters). This would suggest that the ferrets’ behaviour is in fact consistent with a model in which there are four fixed criterion positions corresponding to each of four respective possible outcomes on the previous trial. Perhaps surprisingly, this nevertheless reproduces trends in the criterion at the block level.

**Table 4:** Parameters fitted to no-memory model.

Ferret	Trial Format	σ	*c*_1_	*b*_11_	*b*_01_	*b*_10_	*b*_00_	λ
1	Blocks (8)	6.44	2.47	4.13	−5.24	1.45	2.26	0.07
2	Blocks (8)	4.13	2.54	3.05	−2.09	0.49	2.03	0.05

#### Quantitatively comparing the fit to the summary data

The graphical depictions of the full, no-shift, no-decay and no-memory models’ performances in Figs. 6, 7, 8, and 9 respectively, provide a qualitative impression of where the models succeed and fail. Table 5 provides quantitative measures to accompany these figures, namely, the mean square distance between corresponding empirical and model data points in panel A, and the mean square distance between corresponding empirical and model data points in panel B. For both measures and for both ferrets, the no-shift and no-decay models perform consistently worse than the full model and the no-memory model. The no-shift model slightly outperforms the no-decay model when the comparison is based on panel A. However, the no-decay model outperforms no-shift model when the comparison is based on panel B (ROC space). This may be partly due to the fact that the no-decay model was explicitly fitted to the data in ROC space. However, it is also clear that the fit to the data in **Fig.** 7B is very poor (as it must in principle be). Finally, despite having one less parameter, the no-memory model performs only very slightly worse than the full model. This is of course consistent with the high values of *a* (≥0.85) in the full model. It is also interesting that this model has the same number of parameters as the no-decay model.

**Table 5:** Quality of fit to summary data.

Ferret	TrialFormat	Blocks				ROC			
		full	no shift	no decay	no memory	full	no shift	no decay	no memory
1	Blocks (8)	0.033	0.062	0.067	0.035	0.066	0.236	0.116	0.068
2	Blocks (8)	0.047	0.057	0.060	0.048	0.048	0.179	0.072	0.043

#### Retrospectively fitting psychometric data from other ferrets

The original motivation for this work as to see if we could relate trial-by-trial criterion shifts and criterion shifts observed across entire behavioural sessions that were observed in the course of normal psychoacoustic tests in ferrets. It is therefore relevant to know if the model(s) have explanatory power in the context of more typical test settings.

**Figure 10:**
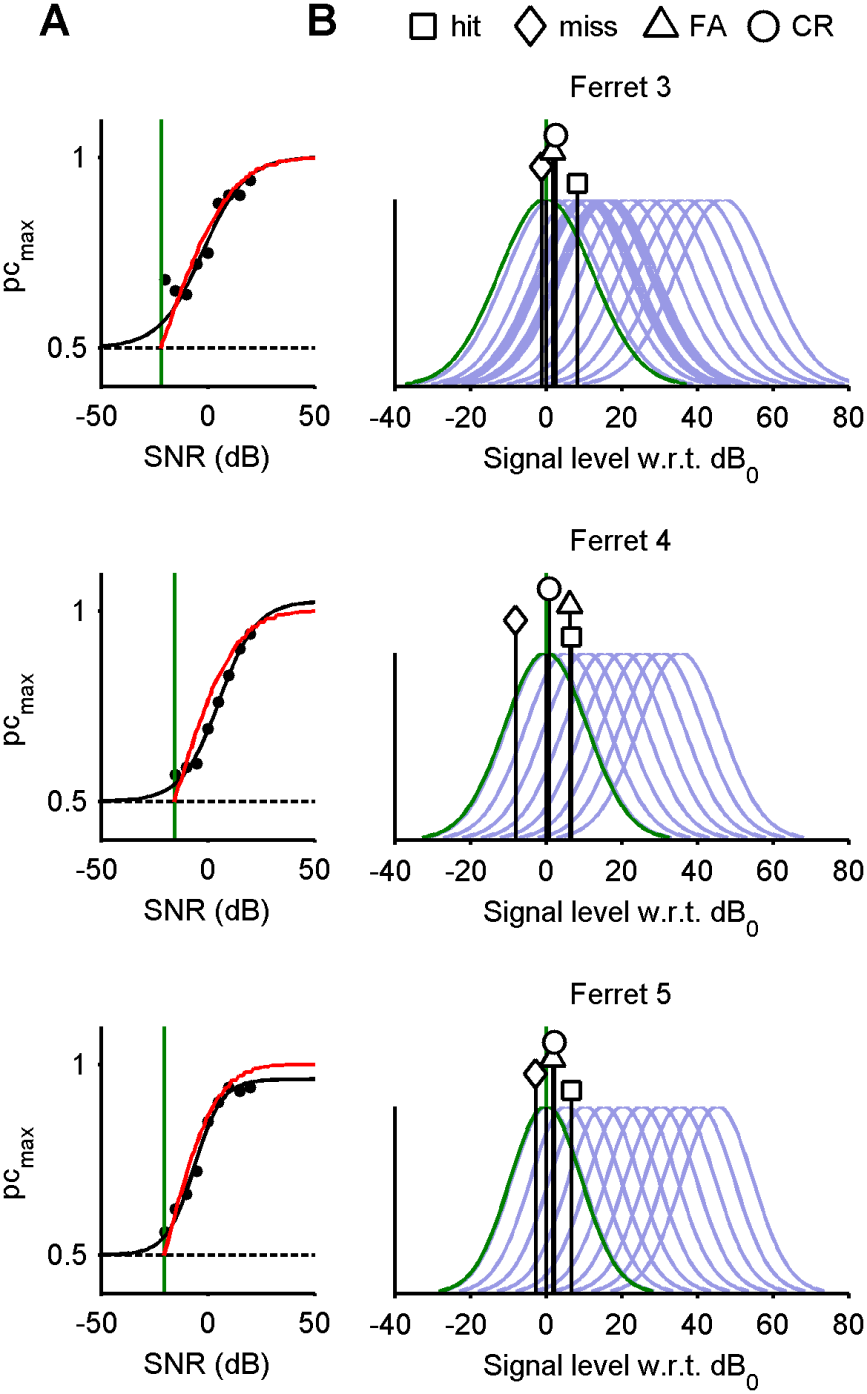
Maximum likelihood parameters fitted to data from ferrets 3, 4 and 5. A) Sigmoid functions (curve) fitted to psychometric data (markers). The reference level (dB_0_) is read off at 5% the height of the curve and is shown as a green vertical. The chance line is dashed. Each red curve plots the theoretical psychometric function that results if criterion shifts are removed from the respective fitted models. B) Four fixed threshold positions (*b_ij_*) corresponding to the four previous outcomes (i.e., full decay, *a* = 1 followed by a shift relative to *c*_1_ = 0). No-signal and signal densities are plotted in green and light blue, respectively.

Fig. 10 displays parameters fitted to trial data collected for three other ferrets (3–5). These data were originally collected to measure psychometric functions [2]. Half the trials were signal trials, with the signal levels drawn randomly with replacement from a set of levels with no serial correlation. However, in the case of an incorrect answer the exact same trial was repeated (i.e. a correction trial; correction trials were not included in any derived measures of performance). This means that certain combinations of outcomes were precluded (a false alarm followed by hit, for example). Furthermore, these data were not collected with this model in mind but to measure the tone level corresponding to a threshold level of performance (usually *d*’ = 1). Thus no effort had been made to ensure that the signal levels straddled the linear portion of the psychometric function. Both of these factors place our method at something of a disadvantage, making it interesting to discover whether the procedure would nonetheless fit the data successfully.

Fig. 10A plots the psychometric functions recovered for ferrets 3 to 5 (excluding the outcomes on correction trials) as black curves. These curves were used to derive reference levels against which signal decibels levels were expressed. The full model, with all parameters, was used. In all three cases, the maximum likelihood fitting routine converged to a solution in which the decay parameter, *a* = 1. This coincides with the no-memory model in which there are four fixed criterion positions corresponding to each of four respective possible outcomes on the previous trial. These four criterion positions are plotted in Fig. 10B (assuming *c*_1_ = 0), along with the signal levels and distribution widths. As before, the most liberal criterion followed misses; and, in the cases of ferrets 3 and 5, the most conservative criterion position followed hits. In the exceptional case of ferret 4, the most conservative criterion followed a false alarm (which is of course a sensible policy, albeit at odds with those of the other four ferrets). Each of the panels in Fig. 10A also includes the theoretical psychometric function (red curve), which would be obtained if the ferret followed the model fit, but adopted a single criterion. The performance of this no-shift theoretical model using one criterion value very slightly improves upon that of the no-memory model using four criteria. A shifting criterion is only expected to lead to performance gains if there are serial correlations in the trial levels, which, in this case, there are not. These results demonstrate that the fitting procedure operates adequately on data which has not been ideally conditioned. It also further supports that the ferrets’ behaviour correspond to a “no-memory” model of dynamic criterion setting.

#### Predictability of decisions

Having examined a model fit at the summary level for two ferrets (see above), we now compare the actual decisions of the model with those of the ferret and measure how often the two agree. The model’s decision on trial *n* is made by comparing the signal level at that trial to the criterion *c_n_*, which in turn is estimated on the basis of the outcomes of all the ferret’s decisions up to and including trial *n–1*. We consider maximum likelihood fits for two alternative model types: the no-shift model, which employs a static criterion, and the full model, in which the criterion shifts on the basis of the preceding trial outcome and decay decays towards to a resting criterion.

Table 6 lists how many decisions the no-decay and full models are capable of predicting for six experiments using five ferrets. In all six cases, the fraction of predictable trials increases by a small margin when the criterion is adaptive. This is of course a very stiff test of whether *every decision* can be predicted, which is impossible given a noisy internal representation, even if the model captured the behaviour perfectly. In four of these cases, the increase is highly significant (*p*<0.0005, one-sided sign rank test). The failure of the full model to predict the decisions of ferret 2 in the blocked-trial task significantly better than the baseline model may be due to the smaller sample size for this ferret, combined with the fact that ferret 2 does not appear to shift its criterion substantially enough to create a large effect size. Despite the freedom the full model has to shift the criterion, the maximum likelihood parameters produce an almost static criterion on average (see Fig. 6B, ferret 2). This in turn may be due to the fact that the levels chosen for ferret 2 were easier to detect than those for ferret 1, making criterion shifts difficult to detect. Besides these explanations, it is possible that this model is simply a poor description of the ferret’s behaviour. That said, there is little doubt that the next response is significantly affected by outcome of the previous one (see Fig. 4), and that the probability of a false alarm is significantly lower during the difficult (even) blocks, as discussed in the Empirical Results section above. Furthermore, the full model does predict the responses of ferret 2 significantly better than the baseline model when the trials are ordered randomly, even given the smaller sample size, perhaps indicating that the randomness of the trials contributes to a wider variation in the criterion.

**Table 6:** Next-trial predictive capacity of the no-shift and full models.

Ferret	Trial Format	# trials	# yes	# predicted	# predicted	Sign rank *p*
				no-shift	full	
1	Blocks (8)	6559	3058	4372	4840	0.000
2	Blocks (8)	2813	1359	2251	2277	0.042
2	Random	816	443	645	668	0.006
3	Random + CT	16366	7494	12870	13108	0.000
4	Random + CT	11163	5444	8453	8927	0.000
5	Random + CT	17566	8114	14319	14605	0.000

## Discussion

Five ferrets were trained to perform a yes-no detection task. Ferrets 1 and 2 were required to detect tones presented in blocks of trials whose difficulty alternated between easy and hard set of levels. For both ferrets, the false alarm probability is significantly lower during the hard blocks. From a signal detection theory perspective this means that a more liberal threshold criterion has been applied during blocks of hard trials, as the false alarm statistics are derived from trials on which no signal was presented. False alarm rates shifted rapidly with each new block, indicating that the criterion is adjusted on the basis of recent signal levels. Furthermore the false alarm probability for the first trial of a block (whether easy or hard) does not differ significantly to that of the last trial in the preceding block, as there has not yet been a signal trial to establish the new context, which argues in favour of adaptation being driven by changes in the outcome of preceding trials.

Our data also show that the outcome of one trial exerts a robust statistical effect on the hit and false alarm probability of the next trial, which in turn reveals an effect on the criterion, making it natural to inquire whether the accumulation of these outcome-driven criterion changes could account for the average, long-term criterion variation observed over whole blocks. Gaining an intuition for how the criterion evolves over many trials is challenging, because the criterion positions and trial outcomes mutually interact. Fortunately, in our model, the criterion dynamics are stationary, making them susceptible to Markov analysis. Formalising the criterion changes as a Markov process allows us to assign a probability to a sequence of decisions to form a likelihood function; moreover, the likelihood function varies smoothly with the model parameters, providing the tractability needed for a maximum likelihood method based on gradient ascent. This fitting method is also advantageous in that it did not seek to explicitly fit the criterion shifts. Rather it attempted to predict the sequences of decisions made. The model fits nevertheless show robust block and trial level criterion shifts, and this reproduces traits of the data better than a model without any dynamic criterion shifts. The full dynamic model demonstrated both trial-by-trial and block level shifts in criterion suggesting that criterion setting could be accounted for by a simple dynamic model in which criterion shifts depending on the outcome of individual trials.

We fitted parameters to six sets of experimental data from five ferrets: two sets of trials were grouped in 8-trial blocks (ferrets 1 and 2); one set of trials used the same four levels but presented them in a randomised order (ferret 2); three sets of trials were psychometric data that used many levels and included correction trials (ferrets 3–5). In all six instances of parameter fitting, the outcome associated the greatest criterion shift in the liberal direction was a miss; in five out of six instances, the outcome that led to the greatest criterion shift in the conservative direction was a hit. This is contrary to the expectation that errors would be most responsible for modifying the criterion–that is, that false alarms would raise the criterion on no-signal trials, just as misses lower it on signal trials. Nevertheless, these parameters comport with the model-free presentation of the data in Fig. 4, which shows that, for both ferrets, and for both easy and hard stimulus levels, false alarms are less likely following hits (square markers) than false alarms (triangular markers). The lack of symmetry between the effect of false alarms and misses, observable both in the data and the model’s account of it, is less surprising upon considering that, although false alarms and misses are symmetrically opposite in a theoretical sense, they are not opposite in natural setting, as they may carry different costs.

**Figure 11:**
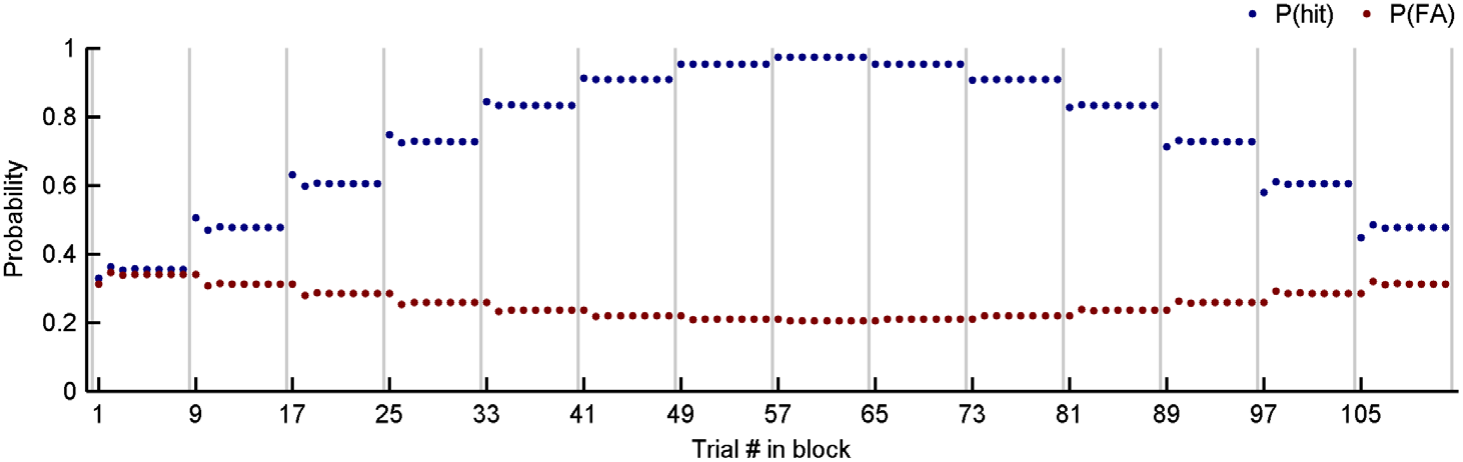
Hit and false alarm for model with full criterion decay. Analytical probability of hit (blue) and false alarm (red) for a model based on the maximum likelihood fit parameters for ferret 4 (*a* = 0, see fit in Fig. 10B, ferret 4). The model is presented with the stimulus levels shown in Fig. 10B in blocks of 8 trials (50% probability of no-signal), ascending from the hardest to easiest level, and back down again. Note that *P*(FA) falls and rises again *smoothly*, and that a small oscillation accompanies the start of each block, which is most noticeable when the signal level is low (e.g., trials 1–3, 9–11).

An additional source of criterion movement, besides outcomes, was a general decay in the direction of a resting criterion (*c*_1_). When the decay parameter, *a*, was a free variable in the fitting procedure, it tended to converge to values close, or equal to, one. Indeed, a memoryless model, in which *a* = 1, performed almost as well as the full model. As it happens, this is not unreasonable in light of the data, which show that a rapid adaptation accompanies the onset of a new block, on the order of one trial (Fig. 1 and 2). There are also other compelling reasons to think that a rapid decay better accounts for long-term dynamics. Firstly, the ferret experiences an ecologically-determined range of stimulus levels during its lifetime, rather than an unbounded one. Whilst the additive model has a theoretical advantage, in that it can position the criterion arbitrarily, the decaying criterion model naturally maintains a default sensitivity to the environment. Secondly, we counter the claim that if *a* = 1, the criterion can assume only four positions, implying that the ferret cannot exhibit an interesting repertoire of behaviour. We do so by noting that the model can still exhibit a continuous range of hit and false alarm probabilities through an equilibrium established through an interaction with the stimulus level. Fig. 11 shows the hit and false alarm probabilities that arise as the signal level is progressively raised and then lowered through 8-trial blocks, according to a model using the parameters fit to the data from ferret 4 (in which *a* = 1). Notice that, although the criterion can only assume four positions, the false alarm probability (which does not depend on the signal level) occupies a continuous range of values. This is because the false alarm (and hit) probabilities are based on the *average* of four criteria, and this average depends on the signal level in the block.

The inclusion of the decay parameter, *a*, whether rapid as in the full model or complete as in the memoryless model, introduces an important degree of freedom into the model, which appears to be demanded by the data. This point concerns the time course of adaptation of P (hit) and P(FA) following the onset of a new block, and the asymptotic values that those quantities reach. In the additive model, the time course and asymptotes are determined, and *coupled* to each other, by the incremental parameters, *b_ij_*. This is evident in Fig. 8A, where there appears to be a compromise between fitting the time course and asymptotes: for ferret 1, the parameters poorly capture the asymptotes; for ferret 2, they poorly capture the time course. Fitting the additive model to the ROC data shown in Fig. 8B is also problematic: the *b_ij_* have to be chosen so that most of the markers line up around a single false alarm rate (≈0.2), with the exception of one outcome which has a substantially higher false alarm probability (misses, diamond markers). The additive model can position the miss marker to the right by associating a large negative shift with a miss (*b*_01_<<0). To align the other markers then requires that the remaining *b_ij_* collectively compensate by encoding carefully-balanced positive shifts, but the markers do not always have the freedom to move to the positions represented by the empirical data, and the result is a compromise. A model which does not simply accumulate criterion shifts decouples the time courses from the asymptotes to some extent, improving the fit to the ROC data, whether it is viewed as conditional upon the position of the trial within a block (Fig. 6A) or the preceding trial (Fig. 6B).

Although the model accounts quite well for the block and trial level shifts, and it is clear that different variations in the model (full, no-shift, no-decay, no-memory) differ in how well they to fit the data, there may of course be alternative models. One worth consideration is whether since predictable blocks of stimulus levels switched at predictable times, ferrets’ could instead switch criterion in discrete steps at the beginning of a new block. The fact that the false alarm probability for the first trial of a block (whether easy or hard) does not differ significantly to that of the last trial in the preceding block, might argue against ferrets learning the block length. However, a model in which criterion was explicitly shifted block by block would then have to explain how hit and false-alarm probabilities which depended asymmetrically on the outcome of the previous trial *did not* also produce criterion shifts at the block level. The model we used here is appealing in that it parsimoniously explains the block level criterion as emerging as a consequence of the empirically observed trial-by-trial shifts.

Classical application of SDT does not distinguish between a broad sensory representation and variability in the decision criterion. Any dynamic changes in criterion will result in shallower psychometric functions. SDT would ordinarily account for this as increased variability in the sensory representation. This can present a challenge when interpreting psychophysical data in general, but perhaps particularly in non-human animals where basic tasks can be cognitively challenging [35]. In our data, the ability to adapt if stimulus statistics change (i.e. between blocks) implies a cost when the task contingencies remain static (within blocks). This is evident in larger σ values fitted to the no-shift model (c.f. Table 2 and Table 1), but the difference is slight, suggesting that performance and thus obtained rewards suffers little as a result of the criterion shifts. So it appears that the ability to adapt to changes does not impact seriously on performance. Neither does it strongly influence the estimation of thresholds in psychophysical performance (unlike in other work, [36]). This finding is in agreement with Alves-Pinto et al. [2], despite the more sophisticated model used here. Neither did inattention, modelled using a lapse probability, have much influence. This does not of course rule out the possibility that there are other sources of decision variability which are influencing performance.

An interesting question is how the specific task the ferrets performed influenced the strategy they adopted. One important component of the task is the use of correction trials. These are necessary in general to instruct errors, particularly during early training, and control response bias (see [2]). Correction trials would tend to encourage an “alternating” strategy since following a mistake the correct answer must be to make the alternative response. Correction trials were omitted during blocked trials so that we could observe criterion shifts in independent trials following errors. However, fitted models gave very similar parameters in sessions with unblocked sessions where the correction trials were included. It seems quite likely therefore that training with correction trials impacts on the ferrets’ strategy and they do not greatly alter their strategy when it is removed. We note that shifts following false alarms, which correction trials would encourage, were small whilst large shifts were observed following hits. Also, regardless of the cause, the models suggest that this shift strategy is effective in optimising the criterion at a block and session level. However, determining whether the observed strategies are driven by training with correction trials or to optimise criterion to differing stimulus sets would require a different training regime.

The methods described here may constitute a framework that is applicable in a wide range of psychophysical experiments, and the Appendices detail the mathematics used. Simple decision dynamics models might explain sequential effects in other species (e.g., humans) or other types of psychological experiments (vision, memory). The model we used here was inherently flexible. Different model parameters, resulting in different shift strategies, can serve to optimise the decision criterion (see Fig. 5), and related models have been fit to human data (without correction trials) previously [3]. However, it remains to be seen whether and how such models would need to be modified to fit a broader range of psychophysical data.

There are two complementary reasons for seeking to extend SDT models to explain the criterion setting process. One reason is to improve the way that psychophysical data is interpreted in studying sensory processing [35]. A fundamental problem in neuroscience how to relate neural processing to perception. There has been a great deal of progress made in recent years in how different aspects of neural responses relate to perceptual limits (e.g. [37]). It is desirable to obtain the physiological and psychophysical data in the same species, but the interpretation of the psychophysics is less often scrutinised. The application of classical SDT in animal psychophysics is still a subject of debate, and classical SDT measures of sensory discrimination are unfortunately not insensitive to cognitive factors [38], [39]. The application of more sophisticated models that do not assume that the decision process is static of may help to better isolate the contribution of perceptual limits in situations where non-sensory factors are not negligible (e.g. [35], [36]).

The other reason to study criterion setting processes is to further understand the basis of decision making. Decision making is more usually studied in situations where the task contingencies are uncertain, rather than the sensory stimulus itself (i.e. sensory stimuli are all suprathreshold). Such work has shown that people and animals are able to assimilate information about a changing environment (or task) and adapt their behaviour optimally over time [24], [40], [41]. It has also shown that simple adaptive rules can explain this behaviour, [23], [28], [41], [42], and that such models may have some physiological basis [26], [27], [43]. Fewer studies have attempted to model the dynamics of decision making in conditions of sensory uncertainty [36] or when stimulus salience and the value of a decision are both varied [25]. Overall, however, evidence from a range of studies including our own suggests that at least in the laboratory, complex problems of optimising decision processes in the face of a changing environment may be solved in practice by very simple dynamic processes and that considering such processes allows for greater insights into sensory processing and decision making.

## Supporting Information

S1 Supplementary Material **Computing the Markov Solution to a Simple Model.**
(PDF)

S2 Supplementary Material **Ascending the Likelihood Function.**
(PDF)

## References

[1] Wenger MJ, Rasche C (2006) Perceptual learning in contrast detection: presence and cost of shifts in response criteria. Psychon Bull Rev 13:656–661. 17201366 10.3758/bf03193977

[2] Alves-Pinto A, Sollini J, Sumner CJ (2012) Signal detection in animal psychoacoustics: analysis and simulation of sensory and decision-related influences. Neurosci 220:215–227. 10.1016/j.neuroscience.2012.06.001 PMC3422536 22698686

[3] Dorfman DD, Biderman M (1971) A learning model for a continuum of sensory states J Math Psychol. 8:264–284.

[4] Thomas EAC (1975) Criterion adjustment and probability matching. Percept Psychophys 18:158–162.

[5] Stuttgen MC, Yildiz A, Gunturkun O (2011) Adaptive criterion setting in perceptual decision making. J Exp Anal Behav 96:155–176. 21909162 10.1901/jeab.2011.96-155 PMC3168885

[6] Baum WM (1981) Optimization and the matching law as accounts of instrumental behavior. J Exp Anal Behav 36:387–403. 16812255 10.1901/jeab.1981.36-387 PMC1333108

[7] Green DM, Swets JA (1966) Signal Detection Theory and Psychophysics. New York: John Wiley & Sons, Inc.

[8] Whalen AD (1971) Detection of signals in nose. New York: Academic Press.

[9] MacMillan NA, Creelman CD (2005) Detection Theory: a User’s Guide. New Jersey: Lawrence Erlbaum Associates.

[10] Frund I, Haenel NV, Wichmann FA (2011) Inference for psychometric functions in the presence of nonstationary behavior. J Vis 11:1–19. 10.1167/11.6.16 21606382

[11] Jesteadt W, Luce RD, Green DM (1977) Sequential effects in judgments of loudness. J Exp Psychol Hum Percept Perform 3:92–104. 845558 10.1037//0096-1523.3.1.92

[12] Verplanck W, Cotton JW, Collier GH (1953) Previous training as a determinant of response dependency at the threshold. J Exp Psychol 46:10–14. 13069661 10.1037/h0050386

[13] Jones P (2013) Mechanisms of auditory perceptual learning: University of Nottingham.

[14] Dorfman DD, Saslow CF, Simpson JC (1975) Learning models for a continuum of sensory states reexamined. J Math Psychol 12:178–211.

[15] Hack MH (1963) Signal detection in the rat. Science 139:758–760. 13951669 10.1126/science.139.3556.758

[16] Blough DS (1967) Stimulus generalization as signal detection in pigeons. Science 158:940–941. 6054168 10.1126/science.158.3803.940

[17] Clopton BM (1972) Detection of increments in noise intensity by monkeys. J Exp Anal Behav 17:473–481. 4624513 10.1901/jeab.1972.17-473 PMC1333924

[18] Marston HM (1996) Analysis of cognitive function in animals, the value of SDT. Brain Res Cogn Brain Res 3:269–277. 8806028 10.1016/0926-6410(96)00012-2

[19] Talwar SK, Gerstein GL (1999) A signal detection analysis of auditory-frequency discrimination in the rat. J Acoust Soc Am 105:1784–1800. 10089602 10.1121/1.426716

[20] Fernberger SW (1920) Interdependence of judgements within the series for the method of constant stimuli. J Exp Psychol 3:126–150.

[21] Fechner W (1860) Elemente der Psychophysik. Leipzig: Breitkopf und Härtel.

[22] Green DM (1964) Consistency of Auditory Detection Judgments. Psychol Rev 71:392–407. 14208857 10.1037/h0044520

[23] Nassar MR, Wilson RC, Heasly B, Gold JI (2010) An approximately Bayesian delta-rule model explains the dynamics of belief updating in a changing environment. J Neurosci 30:12366–12378. 20844132 10.1523/JNEUROSCI.0822-10.2010 PMC2945906

[24] Brunton BW, Botvinick MM, Brody CD (2013) Rats and humans can optimally accumulate evidence for decision-making. Science 340:95–98. 23559254 10.1126/science.1233912

[25] Navalpakkam V, Koch C, Rangel A, Perona P (2010) Optimal reward harvesting in complex perceptual environments. Proc Natl Acad Sci U S A 107:5232–5237. 20194768 10.1073/pnas.0911972107 PMC2841865

[26] Barraclough DJ, Conroy ML, Lee D (2004) Prefrontal cortex and decision making in a mixed-strategy game. Nat Neurosci 7:404–410. 15004564 10.1038/nn1209

[27] Sugrue LP, Corrado GS, Newsome WT (2004) Matching behavior and the representation of value in the parietal cortex. Science 304:1782–1787. 15205529 10.1126/science.1094765

[28] Wilson RC, Nassar MR, Gold JI (2013) A mixture of delta-rules approximation to bayesian inference in change-point problems. PLoS Comput Biol 9:e1003150. 23935472 10.1371/journal.pcbi.1003150 PMC3723502

[29] Treisman M, Williams TC (1984) A theory of criterion setting with an application to sequential dependencies. Psychol Rev 91:68–111.

[30] Kac M (1962) A note on learning signal detection. IRE Trans Inf Theory IT-8:126–128.

[31] Wichmann FA, Hill NJ (2001) The psychometric function: I. Fitting, sampling, and goodness of fit. Percept Psychophys 63:1293–1313. 11800458 10.3758/bf03194544

[32] Klein SA (2001) Measuring, estimating, and understanding the psychometric function: a commentary. Percept Psychophys 63:1421–1455. 11800466 10.3758/bf03194552

[33] Grimmett GR, Stirzaker DR (1992) Probability and Random Processes Oxford: Claredon Press. 541 p.

[34] Bishop CM (2007) Pattern Recognition and Machine Learning. Berlin: Springer. 738 p.

[35] Carandini M, Churchland AK (2013) Probing perceptual decisions in rodents. Nat Neurosci 16:824–831. 23799475 10.1038/nn.3410 PMC4105200

[36] Busse L, Ayaz A, Dhruv NT, Katzner S, Saleem AB, et al (2011) The detection of visual contrast in the behaving mouse. J Neurosci 31:11351–11361. 21813694 10.1523/JNEUROSCI.6689-10.2011 PMC6623377

[37] Jacobs AL, Fridman G, Douglas RM, Alam NM, Latham PE, et al (2009) Ruling out and ruling in neural codes. Proc Natl Acad Sci U S A 106:5936–5941. 19297621 10.1073/pnas.0900573106 PMC2657589

[38] Alsop B (1998) Receiver operating characteristics from nonhuman animals: Some implications and directions for research with humans. Psychon Bull Rev 5:239–252.

[39] Blough DS (2001) Some contributions of signal detection theory to the analysis of stimulus control in animals. Behav Processes 54:127–136. 11369465 10.1016/s0376-6357(01)00154-1

[40] Gallistel CR, Mark TA, King AP, Latham PE (2001) The rat approximates an ideal detector of changes in rates of reward: Implications for the law of effect. J Exp Psychol Anim Behav Process 27:354–372. 11676086 10.1037//0097-7403.27.4.354

[41] Behrens TE, Woolrich MW, Walton ME, Rushworth MF (2007) Learning the value of information in an uncertain world. Nat Neurosci 10:1214–1221. 17676057 10.1038/nn1954

[42] Corrado GS, Sugrue LP, Seung HS, Newsome WT (2005) Linear-Nonlinear-Poisson models of primate choice dynamic. J Exp Anal Behav 84:581–617. 16596981 10.1901/jeab.2005.23-05 PMC1389782

[43] Daw ND, Doya K (2006) The computational neurobiology of learning and reward. Curr Opin Neurobiol 16:199–204. 16563737 10.1016/j.conb.2006.03.006

