# Supplementary material for: Decision Criterion Dynamics in Animals Performing an Auditory Detection Task
Source: PLoS One. 2014 Dec 8;9(12):e114076. doi: 10.1371/journal.pone.0114076 (PMC4259461; doi:10.1371/journal.pone.0114076)
Supplement: S1 Supplementary Material — Computing the Markov Solution to a Simple Model. (PDF) [file pone.0114076.s001.pdf]

Supplementary Material S1:

Markov Solution to a Simple Model

Consider a model in which $c_1 = 0$ and $\sigma = 1$. Let the no-signal trial be associated with mean $\mu_0 = 0$ and the signal trial be associated with mean $\mu_1 = 1$. Assume that corrective shifts of unit magnitude are applied following errors, i.e., $b_{01} = -1$ and $b_{10} = +1$, and that no shifts are applied in the case of correct responses, i.e., $b_{00} = 0$ and $b_{11} = 0$. In this case, the criterion can only fall on integer values, as shown below.

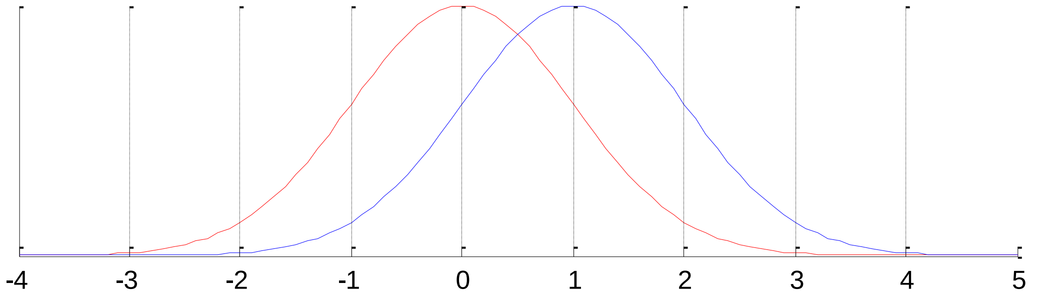

If we assume that the probability of false positives are effectively zero for $c \geq 4$ and the probability of misses are effectively zero for $c \leq 3$, as the figure above suggests, then we can restrict the set of criterion values to $c_n \in \{-3, \ldots, 4\}$.

Transition Matrix

The criterion transitions can be written as

$$\begin{bmatrix} P(c_{n+1} = -3) \\ \vdots \\ P(c_{n+1} = 4) \end{bmatrix} = \begin{bmatrix} P(c_{n+1} = -3 \mid c_n = -3) & \cdots & P(c_{n+1} = -3 \mid c_n = 4) \\ \vdots & \ddots & \vdots \\ P(c_{n+1} = 4 \mid c_n = -3) & \cdots & P(c_{n+1} = 4 \mid c_n = 4) \end{bmatrix} \begin{bmatrix} P(c_n = -3) \\ \vdots \\ P(c_n = 4) \end{bmatrix}$$

or, more compactly, as

$$f_{n+1} = M_n f_n$$

where

$$f_n = \begin{bmatrix} P(c_n = -3) \\ \vdots \\ P(c_n = 4) \end{bmatrix}$$

$$M_n = \begin{bmatrix} P(c_{n+1} = -3 \mid c_n = -3) & \cdots & P(c_{n+1} = -3 \mid c_n = 4) \\ \vdots & \ddots & \vdots \\ P(c_{n+1} = 4 \mid c_n = -3) & \cdots & P(c_{n+1} = 4 \mid c_n = 4) \end{bmatrix}.$$

Note that the probability of transitioning from c_n to c_{n+1} does not depend on the step number. If the probability of a signal is independent of the trial number, then the transition matrix is independent of the step number (that is, $M_n \equiv M$), and the problem can be formulated as a Markov chain:

$$f_{n+1} = M f_n$$

$$f_n = M^{n-1} f_1$$

The elements of M are computed as follows. In the above example upward transitions accompany only false alarms. Similarly, downward transitions accompany only misses. Consequently, for all ξ,

$$P(c_{n+1} = \xi + 1 \mid c_n = \xi) = [1 - P(\text{signal})] \times \frac{1}{\sqrt{2\pi\sigma^2}} \int_{\xi}^{\infty} \exp\left[\frac{(x + \mu_0)^2}{-2\sigma^2}\right] dx$$

$$P(c_{n+1} = \xi - 1 \mid c_n = \xi) = P(\text{signal}) \times \frac{1}{\sqrt{2\pi\sigma^2}} \int_{-\infty}^{\xi} \exp\left[\frac{(x + \mu_1)^2}{-2\sigma^2}\right] dx.$$

The probability that the criterion remains the same from one trial to the next is equal to the probability of a correct decision, that is, for all ξ,

$$P(c_{n+1} = \xi \mid c_n = \xi)$$
$$= \frac{1 - P(\text{signal})}{\sqrt{2\pi\sigma^2}} \int_{-\infty}^{\xi} \exp\left[\frac{(x + \mu_0)^2}{-2\sigma^2}\right] dx + \frac{P(\text{signal})}{\sqrt{2\pi\sigma^2}} \int_{\xi}^{\infty} \exp\left[\frac{(x + \mu_1)^2}{-2\sigma^2}\right] dx.$$

Finally, criterion transitions greater than one are impossible. Consequently, for $k > 1$,

$$P(c_{n+1} = \xi \pm k \mid c_n = \xi) = 0.$$

These possibilities account for all entries in the transition matrix.

Solving the Criterion Distribution

The stationary criterion distribution is found by solving $f^* = Mf^*$. Such a solution can be obtained by computing an eigenvalue decomposition of M and finding the eigenvector corresponding to a unit eigenvalue. Alternatively, analytical solution can be obtained numerically by taking the limit of M^n as $n \to \infty$.

Monte Carlo Trials

The following Matlab code computes the criterion probability mass function for the problem described above by sampling 10,000 random trials.

```
% Reserve space for criteria
N  = 10000;
cn = zeros(N,1);

% Possible criterion positions and initial criterion
cs = -3:4;
c  = 0;

% For each trial n:
for n = 1:N

    % Store criterion
    cn(n) = c;

    % Signal?
    h = rand < 0.5;
    if h

        % Probability yes given signal
        pd = 1 - normcdf(c, 1, 1);

    else

        % Probability yes given no signal
        pd = 1 - normcdf(c, 0, 1);

    end

    % Make decision
    d = rand < pd;

    % Shift criterion
    if d && ~h && c < +4, c = c + 1; end;
    if ~d && h && c > -3, c = c - 1; end;

end

% Compute probability mass
cpmf = hist(cn, cs);
cpmf = cpmf / sum(cpmf);
```

Plotting the criterion results in the following figure (green line and markers).

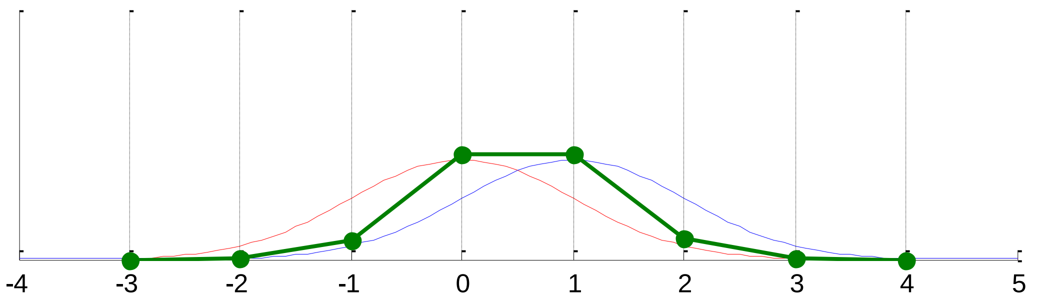

Markov Chain Solution

The stationary criterion distribution is computed analytically using the following code.

```
% Set criterion positions and reserve transition matrix
cs = -3:4;
M  = zeros(length(cs));

% Compute probability of false alarm and miss
p10 = 0.5 * (1 - normcdf(cs, 0, 1));
p01 = 0.5 * normcdf(cs, 1, 1);

% For each criterion position (except edge)
for n = 1:length(cs)-1

    % Compute transition probability
    M(n,n+1) = p01(n+1);
    M(n+1,n) = p10(n);

end

% Compute probability mass analytically
M = M + diag(1 - sum(M,1));
cpmf = null(M - eye(length(cs)));
cpmf = cpmf / sum(cpmf);
```

Plotting the criterion results in the following figure (green line and markers).

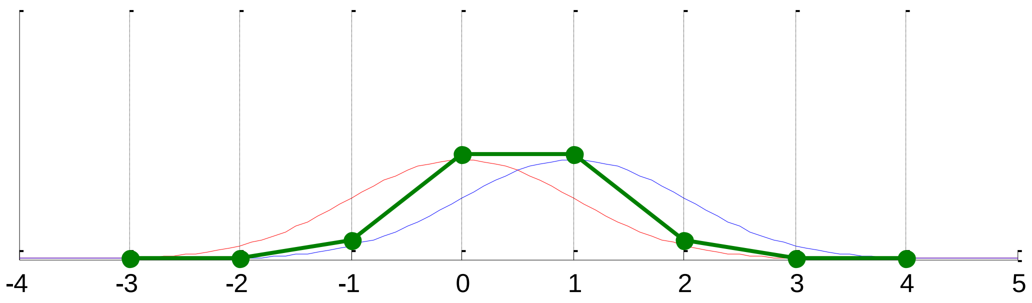

The transition matrix, M, has the following appearance, where darker values represent higher probabilities. Note that upward transitions are more probable when the criterion is low (due to false alarms) and downward transitions are more probable when the criterion is high (due to misses).

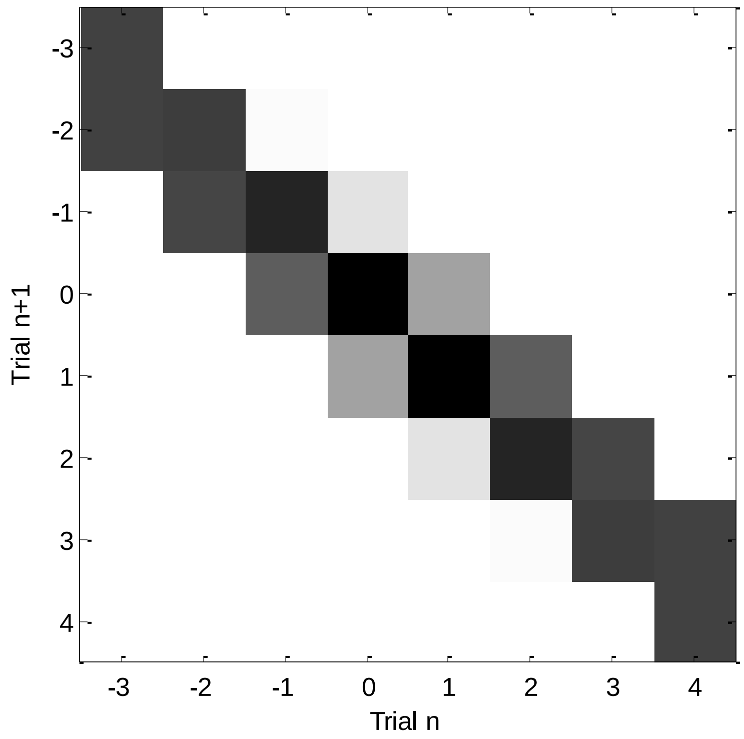

Generalising the Model

This model can be generalised by increasing the number and density of criterion positions to permit more finely graded changes. (The models discussed in the papers use 200 sampling points.) If the criterion shifts to a value which falls between discrete sampling points, a linear interpolation is used to distribute probabilities across the points.
