# Supplementary material for: Decision Criterion Dynamics in Animals Performing an Auditory Detection Task
Source: PLoS One. 2014 Dec 8;9(12):e114076. doi: 10.1371/journal.pone.0114076 (PMC4259461; doi:10.1371/journal.pone.0114076)
Supplement: S2 Supplementary Material — Ascending the Likelihood Function. (PDF) [file pone.0114076.s002.pdf]

Supplementary Material S2:
Ascending the Likelihood Function

Likelihood Function

Probability of a Positive Response

The probability that the internal random decision variable X exceeds the threshold c_n on trial n depends on the current stimulus, h_n, μ_n; the sequences of stimuli and decisions that have preceded trial n, $\mathcal{H}_{n-1} = \{h_1, \dots, h_{n-1}, \mu_1, \dots, \mu_{n-1}\}$, $\mathcal{D}_{n-1} = \{d_1, \dots, d_{n-1}\}$; and the set of model parameters, Θ. We can abbreviate this probability using z_n, then write:

$$
\begin{aligned}
z_n &\equiv \Pr(X \geq c_n \mid \mathcal{H}_n, \mathcal{D}_{n-1}, \Theta) \\
&= \frac{1}{\sqrt{2\pi\sigma^2}} \int_{c_n}^{\infty} \exp\left[\frac{(x-\mu_n)^2}{-2\sigma^2}\right] dx \\
&= \frac{1}{\sqrt{2\pi\sigma^2}} \int_{0}^{\infty} \exp\left[\frac{(x+c_n-\mu_n)^2}{-2\sigma^2}\right] dx.
\end{aligned}
$$

The probability of a positive response (“yes”) is based on z_n, but incorporates the possibility of a lapse. For this we write

$$
\begin{aligned}
y_n &\equiv \Pr(D_n = 1 \mid \mathcal{H}_n, \mathcal{D}_{n-1}, \Theta) \\
&= \frac{\lambda}{2} + (1-\lambda) z_n.
\end{aligned}
$$

Log-likelihood Function

We define the log-likelihood l_n of a decision d_n as the natural logarithm of its conditional probability, which in turn depends on the probability of a positive response:

$$
\begin{aligned}
l_n &\equiv \ln \Pr(D_n = d_n \mid \mathcal{H}_n, \mathcal{D}_{n-1}, \Theta) \\
&= \ln[d_n y_n + (1-d_n)(1-y_n)]
\end{aligned}
$$

The log-likelihood of a decision sequence is then simply the sum of the log-likelihoods of the individual decisions, that is, $\Lambda_n = \sum_{n=1}^{N} l_n$. Finding the maximum likelihood parameters can be achieved by ascending the gradient of Λ_n,

$$\nabla_\Theta \Lambda_n = \sum_{n=1}^{N} \nabla_\Theta l_n = \sum_{n=1}^{N} \frac{(2d_n - 1)\nabla_\Theta y_n}{d_n y_n + (1 - d_n)(1 - y_n)}.$$

Note that the gradient of the log-likelihood function can be expressed in terms of the gradient of the probability of a positive response, $\nabla_\Theta y_n$.

Partial Derivatives of the Likelihood Function

We now consider the partial derivatives with respect to eight parameters that form the components of the gradient $\nabla_\Theta y_n$, namely, those with respect to b_{00}, b_{01}, b_{10}, b_{11}, σ, a, c_1 and λ.

Derivative with respect to Shifts ($\boldsymbol{b_{ij}}$)

The derivative of the probability of a positive response with respect to one of the shift parameters, b_{ij}, is given by

$$\frac{\partial y_n}{\partial b_{ij}} = \frac{1-\lambda}{\sqrt{2\pi\sigma^2}} \int_0^\infty \frac{\partial}{\partial b_{ij}} \exp\left[\frac{(x + c_n - \mu_n)^2}{-2\sigma^2}\right] dx$$

$$= -\frac{1-\lambda}{\sqrt{2\pi\sigma^2}} \frac{\partial c_n}{\partial b_{ij}} \int_0^\infty \frac{(x + c_n - \mu_n)}{\sigma^2} \exp\left[\frac{(x + c_n - \mu_n)^2}{-2\sigma^2}\right] dx$$

$$= -\frac{\partial c_n}{\partial b_{ij}} \frac{1-\lambda}{\sqrt{2\pi\sigma^2}} \exp\left[\frac{(c_n - \mu_n)^2}{-2\sigma^2}\right].$$

The derivative of the criterion, c_n, with respect to the shift parameter, b_{ij}, takes the form of a first-order linear filter applied to the indicator function r_n^{ij}:

$$\frac{\partial c_n}{\partial b_{ij}} = (1-a)\frac{\partial c_{n-1}}{\partial b_{ij}} + \frac{\partial}{\partial b_{ij}}\{a c_0\} + \frac{\partial}{\partial b_{ij}}\left\{\sum_{i,j} r_{n-1}^{ij} b_{ij}\right\}$$

$$= (1-a)\frac{\partial c_{n-1}}{\partial b_{ij}} + r_{n-1}^{ij},$$

where $\frac{\partial c_1}{\partial b_{ij}} = 0$.

Derivative with respect to Standard Deviation (σ)

The derivative of the probability of a positive response with respect to the standard deviation parameter, σ, is given by

$$\frac{\partial y_n}{\partial \sigma} = \frac{\partial}{\partial \sigma}\frac{1-\lambda}{\sqrt{2\pi\sigma^2}}\int_0^\infty \exp\left[\frac{(x+c_n-\mu_n)^2}{-2\sigma^2}\right]dx$$

$$= -\frac{z_n(1-\lambda)}{\sigma} - \frac{2(1-\lambda)}{\sigma\sqrt{2\pi\sigma^2}}\int_0^\infty \frac{(x+c_n-\mu_n)^2}{-2\sigma^2}\exp\left[\frac{(x+c_n-\mu_n)^2}{-2\sigma^2}\right]dx$$

$$= \frac{1-\lambda}{\sqrt{2\pi\sigma^4}}(c_n-\mu_n)\exp\left[\frac{(c_n-\mu_n)^2}{-2\sigma^2}\right].$$

Derivative with respect to Decay (a)

The derivative of the probability of a positive response with respect to the decay parameter, a, is given by

$$\frac{\partial y_n}{\partial a} = \frac{1-\lambda}{\sqrt{2\pi\sigma^2}}\int_0^\infty \frac{\partial}{\partial a}\exp\left[\frac{(x+c_n-\mu_n)^2}{-2\sigma^2}\right]dx$$

$$= \frac{\partial c_n}{\partial a}\frac{2(1-\lambda)}{\sqrt{2\pi\sigma^2}}\int_0^\infty \frac{(x+c_n-\mu_n)}{-2\sigma^2}\exp\left[\frac{(x+c_n-\mu_n)^2}{-2\sigma^2}\right]dx$$

$$= -\frac{\partial c_n}{\partial a}\frac{1-\lambda}{\sqrt{2\pi\sigma^2}}\exp\left[\frac{(c_n-\mu_n)^2}{-2\sigma^2}\right].$$

The derivative of the criterion, c_n, with respect to the decay parameter, a, is then:

$$\frac{\partial c_n}{\partial a} = \frac{\partial}{\partial a}\{(1-a)c_{n-1}\} + \frac{\partial}{\partial a}\{ac_1\} + \frac{\partial}{\partial a}\left\{\sum_{i,j} r_{n-1}^{ij} b_{ij}\right\}$$

$$= (1-a)\frac{\partial c_{n-1}}{\partial a} + c_1 - c_{n-1},$$

for $n > 1$, and $\frac{\partial c_1}{\partial a} = 0$.

Derivative with respect to the Resting Criterion (c_1)

The derivative of the probability of a positive response with respect to the resting (and initial) criterion parameter, c_1, is given by

$$\frac{\partial y_n}{\partial c_1} = \frac{1-\lambda}{\sqrt{2\pi\sigma^2}} \int_0^\infty \frac{\partial}{\partial c_1} \exp\left[\frac{(x + c_n - \mu_n)^2}{-2\sigma^2}\right] dx$$

$$= \frac{\partial c_n}{\partial c_1} \frac{2(1-\lambda)}{\sqrt{2\pi\sigma^2}} \int_0^\infty \frac{(x + c_n - \mu_n)}{-2\sigma^2} \exp\left[\frac{(x + c_n - \mu_n)^2}{-2\sigma^2}\right] dx$$

$$= -\frac{\partial c_n}{\partial c_1} \frac{1-\lambda}{\sqrt{2\pi\sigma^2}} \exp\left[\frac{(c_n - \mu_n)^2}{-2\sigma^2}\right].$$

The derivative of the criterion c_n with respect to the resting criterion parameter (c_1) is then

$$\frac{\partial c_n}{\partial c_1} = \frac{\partial}{\partial c_1}\{(1-a)c_{n-1}\} + \frac{\partial}{\partial c_1}\{ac_1\} + \frac{\partial}{\partial c_1}\left\{\sum_{i,j} r_{n-1}^{ij} b_{ij}\right\}$$

$$= (1-a)\frac{\partial c_{n-1}}{\partial c_1} + a$$

$$= 1,$$

because $\frac{\partial c_1}{\partial c_1} = 1$.

Derivative with respect to the Lapse Probability (λ)

The derivative of the probability of a positive response with respect to the lapse probability parameter, λ, is given by

$$\frac{\partial y_n}{\partial \lambda} = \frac{1}{2} - z_n.$$
